# Experimental verification of strain-dependent relationship between mycovirus and its fungal host

**DOI:** 10.1016/j.isci.2023.107337

**Published:** 2023-07-10

**Authors:** Misa Kuroki, Takashi Yaguchi, Syun-ichi Urayama, Daisuke Hagiwara

**Affiliations:** 1 Faculty of Life and Environmental Science, University of Tsukuba, Tsukuba, Ibaraki 305-8577, Japan; 2 Medical Mycology Research Center, Chiba University, Inohana, Chou-ku, Chiba 260-8673, Japan; 3 Microbiology Research Center for Sustainability, University of Tsukuba, Tsukuba, Ibaraki 305-8577, Japan

**Keywords:** Virology, Mycology

## Abstract

Mycoviruses are viruses that infect fungi. Unlike mammalian infectious viruses, their life cycle does not generally have an extracellular stage, and a symbiosis-like relationship is maintained between virus and host fungi. Recently, mycoviruses have been reported to show effects on host fungi, altering biological properties such as growth rate, virulence, drug resistance, and metabolite production. In this study, we systematically elucidated the effects of viruses on host cells by comparing host phenotypes and transcriptomic responses in multiple sets of virus-infected and -eliminated *Aspergillus flavus* strains. The comparative study showed that mycoviruses affect several cellular activities at the molecular level in a virus- and host strain-dependent manner. The virus-swapping experiment revealed that difference with only three bases in the virus genome led to different host fungal response at the transcriptional level. Our results highlighted highly specific relationship between viruses and their host fungi.

## Highlights

• Mycovirus was explored from clinical and environmental isolates of *Aspergillus flavus*
• Mycoviruses affected fungal cellular properties at the molecular level
• The effects of mycovirus occurred in a virus- and host strain-dependent manner
• Highly specific relationship was suggested between viruses and their host fungi

## Graphical abstract

**Figure:**
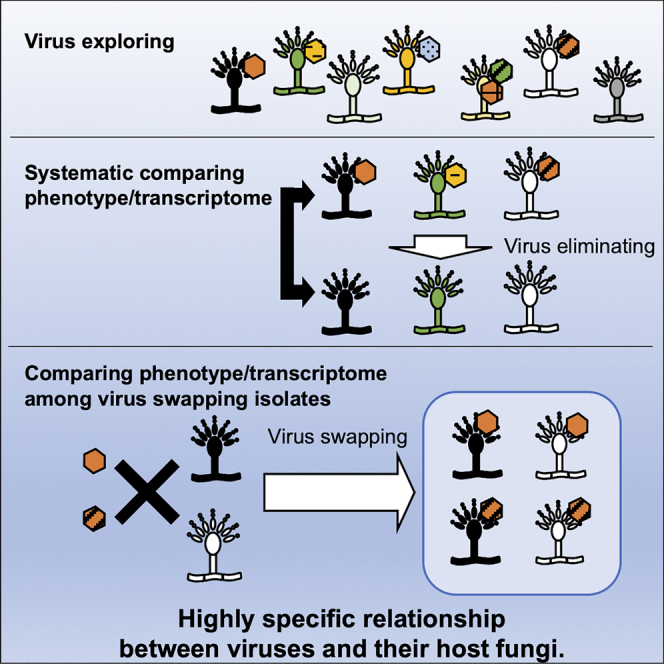

## Introduction

Mycoviruses are viruses that infect fungi. Most mycoviruses are RNA viruses, and they are vertically transmitted to newly generated cell spaces as the cells grow or during cell division. Mycoviruses do not destroy the host cells, and it is thought that they have no extracellular phase in their life cycle and no infection machinery.^1^ Mycoviruses were originally isolated from mushroom. Further, reports of mycovirus isolation have accumulated from species of Basidiomycota, Ascomycota, and Zygomycota, indicating that they are widely distributed across fungal species. The detection of viruses has been advanced by double-stranded RNA (dsRNA)-sequencing and *meta*-transcriptome analysis from environmental samples.^2^^,^^3^^,^^4^ Until now, 267 mycoviruses have been discovered in 81 different fungal species^5^ and recent metagenomic analysis suggests that mycoviruses are present in more fungi than we thought. RNA mycoviruses often have 2–15 kb genome and up to 12 proteins are estimated to be encoded. However, there is little information on viral proteins except for two; RNA-dependent RNA polymerase (RdRp), which replicates the genome itself, and capsid proteins, which make up the viral particles. Thus, the analysis of mycovirus gene function is currently lagging behind that of other viruses that infect animals and plants.

Functional analyses of mycoviruses can involve phenotypic comparison between mycovirus-infected strains and isolates in which the virus has been eliminated. Several viruses are known to affect host biology such as vegetative growth, pathogenicity, metabolite production, and stress tolerance. For example, virus-infected strains showed decreased pathogenicity in *Cryphonectria parasitica*, *Alternaria alternata*, *Aspergillus fumigatus*, and *Pestalotiopsis theae*^6^^,^^7^^,^^8^^,^^9^ and decreased production of secondary metabolites (SMs), e.g., trichothecenes in *Fusarium graminearum*^10^ and deoxynivalenol in *Fusarium pseudograminearum*,^11^ but increased tenuazonic acid in *Magnaporthe oryzae*.^12^ In *Stemphylium lycopersici*, viral infection reduced growth and altersolanol A production. Furthermore, genetic experiments revealed that viral open reading frame 3 (ORF3) suppressed host virulence.^13^ Rosellinia necatrix megabirnavirus 1 and Sclerotinia sclerotiorum hypovirulence-associated DNA virus 1 (SsHADV1) also reported to reduce the host virulence.^14^^,^^15^^,^^16^ In addition, SsHADV1 changed the host fungal property from pathogens to beneficial endophyte. These viral effects may be applicable for the control of plant or human pathogenic fungi in agricultural and clinical settings in the future.^6^^,^^17^^,^^18^^,^^19^^,^^20^ On the other hand, many mycoviruses have no effect on host phenotype under experimental conditions.^21^ Thus, determinants of such viral effects remain an open question despite the many mycoviruses that are increasingly being identified from the meta-data.

In elucidating the role of viruses in fungal ecology, an important challenge has been the elimination of the virus from the host fungus. Some virus-elimination methods have been developed using application of cycloheximide or the antiviral compound ribavirin. In general, however, the frequency of successful virus elimination is dependent on the virus species, and sometimes the removal of certain virus species has been unsuccessful.^22^ This has made it difficult to generate virus-free isolates for single studies of multiple virus species. Recently, our group evaluated the frequency of virus elimination using ten species of viruses and found that the antiviral compound 2′-C-methylcytidine showed greater elimination efficacy than previously used compounds.^23^ This would accelerate virus research, especially for systematic functional analysis of multiple viruses.

Another challenge is the difficulty in extracting general insights from the reported virus effects across fungal or viral species since different strains of host and virus were used in these studies. For example, MoCV1-A and MoCV1-B, which infect different strains of *M. oryzae*, have a high degree of sequence similarity; however, MoCV1-B shows greater effects on melanin biosynthesis, growth rate, and conidiation than MoCV1-A.^24^ The factors that determine these phenotypic differences remain unclear. Therefore, genotypically different hosts within the same fungal species infected by the same virus strain or isogenic hosts infected by different virus strains are required for thorough evaluation of the virus effects on fungal species.

Besides phenotypic evaluation, comparative transcriptome analyses have been conducted between virus-infected and -free isolates in several studies.^10^^,^^11^^,^^25^^,^^26^ These reports demonstrated that mycoviruses drastically affect host gene expression, regardless of the phenotypic changes observed between the virus-infected and -free isolates. Although reports of transcriptomic analyses are accumulating, comparisons of inter-laboratory and different virus-related data have been difficult to make when attempting to extract key factors for virus effects on host cells. Therefore, comparisons using single host species/strains or single viruses are needed to clarify the functions of mycoviruses in host fungi. Thus, in this study, we screened mycoviruses from environmental and clinical isolates of *Aspergillus flavus*, a mycotoxin-producing fungus, and conducted comparative phenotypic and transcriptomic analyses across fungal strains and virus species. Multiple virus-infected strains were found, and isogenic virus-free isolates were generated using antiviral compounds. The fungal phenotypes were comprehensively compared, which revealed that the virus effects differed independently of viral and fungal strains. This finding was also supported by comparative transcriptomic analysis. Unexpectedly, the phenotypic and transcriptomic responses were generally inconsistent even between the virus-swapped isolates, which indicated that the effect of mycoviruses is highly unique to the combination of virus and host genotypes.

## Results

### Mycoviruses identified from *A. flavus* strains

**Figure 1:**
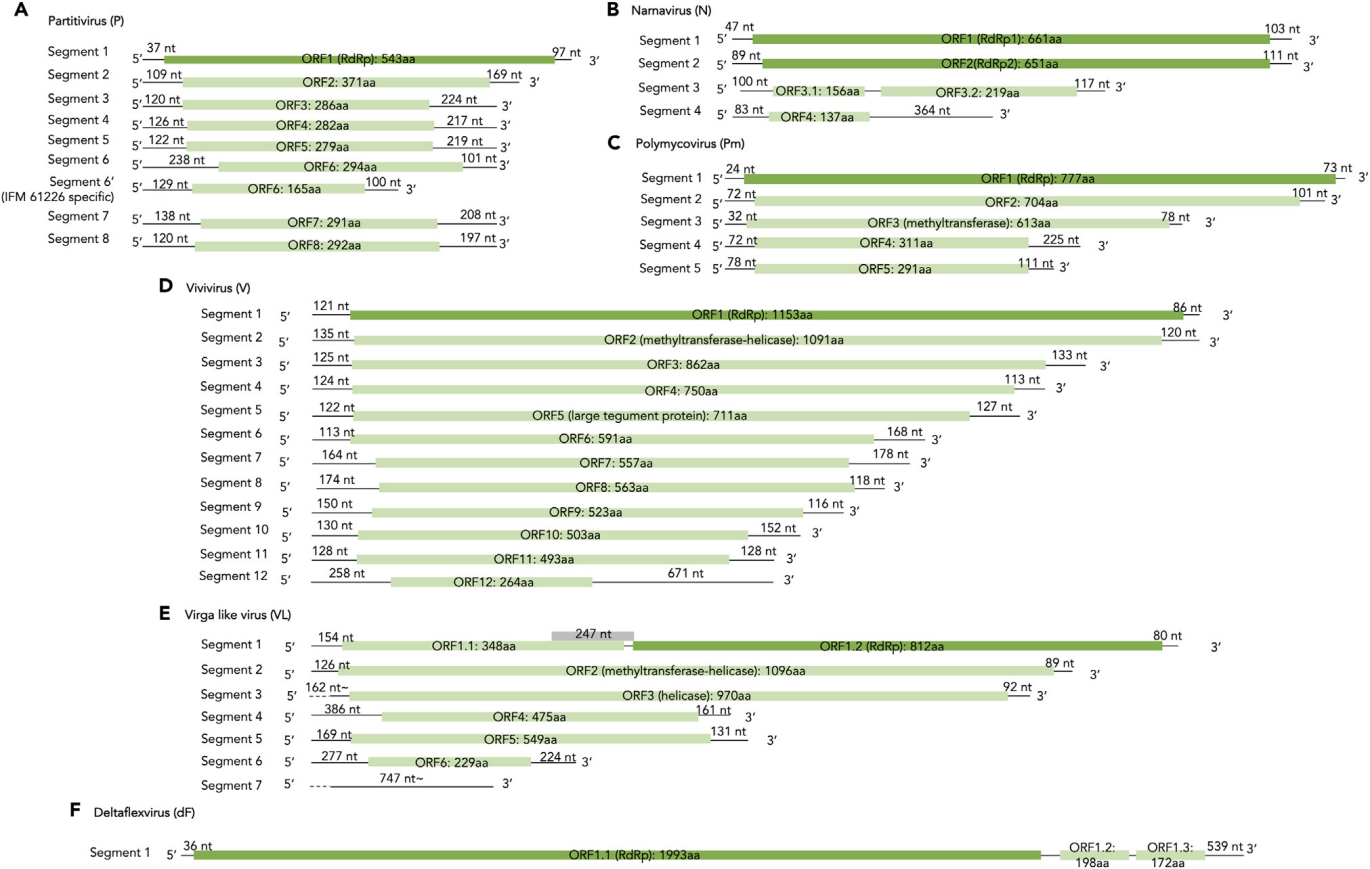
Schematic viral genome structure (A–F) Predicted viral genome structures of partitivirus (A), narnavirus (B), polymycovirus (C), vivivirus (D), virga-like virus (E), and deltaflexivirus (F) are shown. ORFs are predicted in the genomes, which are shown with its amino acid length and the positions of start codon and stop codon. See also Figures S1 and S2, and Table S2.

We screened for mycoviruses in 73 *A. flavus* strains that were isolated from environmental and clinical samples (See Table S1). Nine strains were determined to harbor mycovirus(es) by dsRNA detection, a hallmark of RNA virus infection, in agarose gel electrophoresis (See Figure S1) and dsRNA sequencing by fragmented and primer-ligated dsRNA sequencing (FLDS) (See Materials and Methods). The RNA viruses identified are summarized in Table 1, where virus species were assigned based on sequence similarity. IFM 63847 was infected with multiple viruses, partitivirus and polymycovirus. In total, four partitiviruses, two narnaviruses, one vivivirus, one deltaflexivirus, one polymycovirus, and one unassigned virus were identified from the strain set (Table 1, See also Table S2). The four partitiviruses possessed identical genome structure and predicted ORF contents, except for the partitivirus isolated from IFM 61226, which contained a long deletion in segment 6 (Figure 1A). The genome structure of the narnaviruses infecting the two fungal strains was identical (Figure 1B). The genome sequence of the two narnaviruses was completely identical, whereas several alterations were observed among the partitiviruses sequences.

**Table 1:** List of *Aspergillus flavus* strains and associated mycoviruses

Host accession	Host’s isolation source	Virus name	Virus abbreviation	Blastx top hit					Estimated construction	Estimated molecular type
				Description	E value	Per. ident	Acc. Len	Accession		
IFM 65242	septum, BALF	Aspergillus flavus partitivirus 1	P	RdRp [Aspergillus flavus partitivirus 1]	0.0	84.84%	548	QDE53634.1	35–40 nm/non-enveloped	dsRNA
IFM 65241	septum, BALF	Aspergillus flavus partitivirus 1	P	RdRp [Aspergillus flavus partitivirus 1]	0.0	84.84%	548	QDE53634.1	35–40 nm/non-enveloped	dsRNA
IFM 64473	BALF	Aspergillus flavus deltaflexivirus 1	dF	RNA-dependent RNA polymerase [Aspergillus flavus deltaflexivirus 1]	0.0	98.24%	1992	UAW09565.1	capsidless	ssRNA(+)
IFM 63847	conjunctival sac	Aspergillus flavus partitivirus 1	P	RdRp [Aspergillus flavus partitivirus 1]	0.0	85.03%	548	QDE53634.1	35–40 nm/non-enveloped	dsRNA
		Aspergillus flavus polymycovirus 1	Pm	RNA-dependent RNA polymerase [Aspergillus flavus polymycovirus 1]	0.0	99.48%	776	UAW09573.1	Non-conventionally encapsidated/coated by a viral protein	dsRNA
IFM 63449g	nail plate	Aspergillus flavus narnavirus 1	N	RNA-dependent RNA polymerase [Aspergillus flavus narnavirus 1]	0.0	71.79%	657	UAW09566.1	No true virion, no structural proteins.	ssRNA(+)
IFM 63449w	nail plate	Aspergillus flavus narnavirus 1	N	RNA-dependent RNA polymerase [Aspergillus flavus narnavirus 1]	0.0	71.79%	657	UAW09566.1	No true virion, no structural proteins.	ssRNA(+)
IFM 61879	head (dead body)	Aspergillus flavus vivivirus 1	V	RNA-dependent RNA polymerase [Aspergillus flavus vivivirus 1]	0.0	99.74%	1152	UAW09577.1	Non-enveloped, rigid helical rods with a helical symmetry, about 20–25 nm in diameter with a central “canal”.	ssRNA(+)
IFM 61226	septum	Aspergillus flavus partitivirus 1	P	RdRp [Aspergillus flavus partitivirus 1]	0.0	84.84%	548	QDE53634.1	35–40 nm/non-enveloped	dsRNA
IFM 49866	rice (Vietnam)	Aspergillus flavus virga-like virus	VL	RNA-dependent RNA polymerase, partial [Sisal-associated virgavirus A]	1.00E-25	25.05%	1128	QYJ09848.1	Non-enveloped, rigid helical rods with a helical symmetry, about 20–25 nm in diameter with a central “canal”.	ssRNA(+)

Phylogenetic trees were constructed using the RdRp amino acid sequence of each virus (See Figures S2–S4). As described previously, the sequences of the four partitiviruses are similar to Aspergillus flavus partitivirus 1,^27^ while they are apparently distanced from Aspergillus flavus partitivirus 2.^28^ Notably, the four partitiviruses sequenced by FLDS contained eight segments, which differed from Aspergillus flavus partitivirus 1, which was reported to contain three segments^27^^,^^28^ (See Table S2). In the partitivirus sequenced in this study, segment 1, 2, and 4 showed relatively high homology to the sequence of RNA1, RNA2, and RNA3 of Aspergillus flavus partitivirus 1 (78%–88%) respectively, while segment 3 and 5 showed low homology (30%–39%) to the RNA3 of Aspergillus flavus partitivirus 1 sequence. Segments 6, 7, and 8 have no corresponding sequence in the Aspergillus flavus partitivirus 1 genome.

In addition to partitivirus, the polymycovirus isolated from IFM 63847, deltaflexivirus isolated from IFM 64473, narnaviruses isolated from IFM 63449g and IFM 63449w, and vivivirus isolated from IFM 61879 are most similar to Aspergillus flavus polymycovirus 1, Aspergillus flavus deltaflexivirus 1, Aspergillus flavus narnavirus 1, and Aspergillus flavus vivivirus 1, respectively.^28^ Compared with the reported virus sequences, there were additional segment(s) in the viruses that were sequenced by FLDS, namely, polymycovirus, narnavirus, and vivivirus. They had one, one, and eight novel segments, respectively (Table S2). It is also noteworthy that all of the virus-infecting fungal strains excepting IFM 49866 in this study were isolated in Japan, while the corresponding virus-infecting fungal strains reported in the past were isolated in China (AfPV1) or Italy (the other viruses). This may reflect the situation where the same virus isolates with high identity is distributed worldwide. In fact, virus isolates for Aspergillus flavus deltaflexivirus 1, Aspergillus flavus polymycovirus 1, and Aspergillus flavus vivivirus 1 showed high percentage of identity (98.24%–99.74% in RdRp) between Japan and Italy isolates, whereas Aspergillus flavus partitivirus 1 and Aspergillus flavus narnavirus 1 are relatively diversified (84.84% and 76.75% in RdRp, respectively) (See Table S2).

For the virus of IFM 49866, seven segments were detected, two of which were not fully sequenced. This virus had ORF1 in segment 1, and its sequence showed low homology (25%) to RdRp of Sisal-associated virgavirus A (Table S2). The sequence of ORF1 likely belongs to *virgaviridae* family according to the phylogenetic tree (Figure S4). Therefore, we tentatively named this virus as virga-like virus 1 in this report. Segment 2 of this virus shows homology with methyltransferase of Erysiphe necator-associated virga-like virus 14 (32%), segment 3 has homology with CI protein of Erysiphe necator-associated poty-like virus 2 (31%), and segment 5 has homology with hypothetical protein of Aspergillus fumigatus RNA virus 1 (37%). The other three segments showed no homology to known viral sequences.

Regarding the fungal hosts, the genomes of virus-infected strains were sequenced. A phylogenetic tree was constructed, which revealed that there was a close relationship between IFM 65242 and IFM 65241, and IFM 63449g and IFM 63449w (See Figure S5). The strains IFM 65242 and IFM 65241 were isolated from a single patient one month apart. IFM 63449g and IFM 63449w were derived from a single sample and showed apparently different colony surface color. Therefore, the genetic lineage of these combinations of strains was most likely to be identical.

### Virus effects on host physiology

To gain insights into viral effects on the host, virus-free isolates were established using nucleoside analogs (See Materials and Methods) and confirmed by RT-PCR (See Figure S6). Regarding IFM 63847 co-infected with partitivirus and polymycovirus, single virus-infected strains for each virus were also generated (designated as IFM 63847 Pm or IFM 63847 P infected with polymycovirus or partitivirus, respectively). Unfortunately, growth of the IFM 61226 strain was very slow, and the virus infection was unstable. Therefore, we omitted this strain in further experiments. In all pairs of virus-infected and -free isolates, the colony surface morphology and color were largely indistinguishable (See Figure S7A). We further compared gross morphology under stereomicroscopy, conidiation, UV resistance, and drug resistance (See Figures S7B and S8A–S8C). The virus had no effect on colony growth, mycelial morphology, and drug resistance in any of the strains. Conidiation was reduced by infection with both the partitivirus and polymycovirus in IFM 63847, whereas it was promoted by the virga-like virus 1 in IFM 49866. In IFM 63847 (partitivirus and polymycovirus) and IFM 61879 (vivivirus 1) strains, aerial mycelia formation was altered, and UV resistance of conidia was enhanced by virus infection.

### Effect of viral infection on secondary metabolism

**Figure 2:**
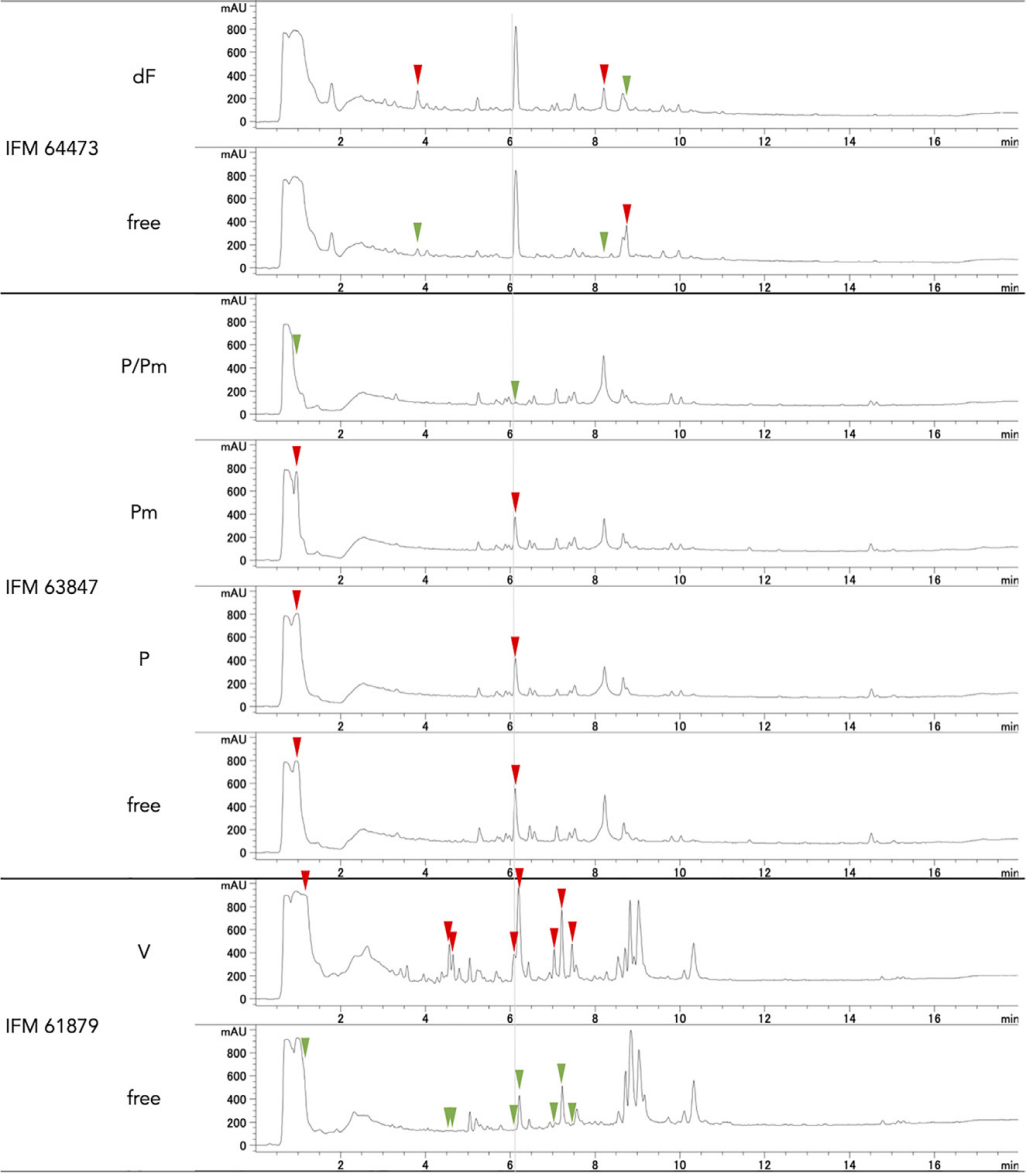
Secondary metabolite profiles of virus-infected and -free isolates Culture extracts of virus-infected and -free isolates of IFM 64473, IFM 63847, and IFM 61879 were analyzed by HPLC. Arrow heads indicate the peak showing increased (red) or decreased (green) metabolite production between virus-infected and -free isolates. Metabolite production was detected at 214 nm. Definitions of virus abbreviations are shown in Figure 1. See also Figure S9.

SM production was investigated in the eight sets of virus-infected and -free isolates. In IFM 64473 (deltaflexivirus), IFM 63847 (partitivirus and polymycovirus), and IFM 61879 (vivivirus 1), SM profiles were significantly altered by virus infection (Figure 2), whereas the other five strains were largely unaffected. Interestingly, production of the compound detected at a retention time of 6.1 min was increased by vivivirus infection in IFM 61879, but decreased by co-infection of partitivirus and polymycovirus in IFM 63847. In IFM 64473, this compound was not produced regardless of virus infection. The production of known mycotoxins of *A. flavus*, aflatoxin (AF) and cyclopiazonic acid (CPA), was investigated (See Figure S9). According to the genome sequences, full-length *afl* cluster exists in IFM 65242, IFM 65241, and IFM 63847 strains or *cpa* gene cluster in IFM 65242, IFM 65241, IFM 63847, IFM 61879, and IFM 49866 strains (See Figure S10). In fact, AF production was detected only in IFM 65242 and IFM 65241, whereas CPA was produced in IFM 65242, IFM 65241, and IFM 61879. Virus infection had no effect on AF or CPA production level in these strains (See Figure S9).

### Comparative transcriptome analysis between virus-infected and -free isolates

**Figure 3:**
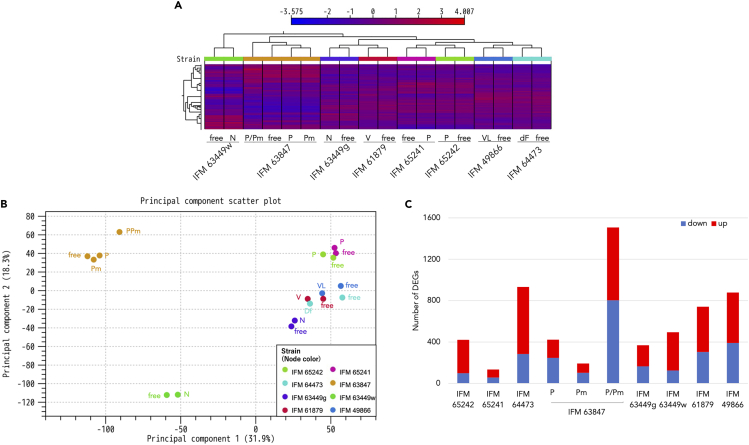
Comparative transcriptome analysis between virus-infected and -free isolates (A) Heatmap of gene expression levels in virus-infected strains and the corresponding virus-free isolates. The heatmap was generated according to TPM data for the whole genome. The color bar shows the range of log2 (TPM) for each gene. A phylogenetic tree presented above the heatmap shows the relationships in expression patterns among samples. (B) PCA plot for gene expression in each isolate. Virus-infected and -free isolates derived from the same strain are shown as the same color. These figures were analyzed and generated by CLC genomics workbench software. (C) Number of differentially expressed genes (DEGs) affected by virus infection in each strain. Genes in which TPM was up- or downregulated >2.5 times by virus infection were regarded as DEGs. Definitions of virus abbreviations are shown in Figure 1. See also Tables S3, S4, and S5.

To obtain deeper insights into virus effects on host biology, we conducted comparative transcriptome analysis between virus-infected and -free isolates for all sets. First, a heatmap was generated with the transcriptome data from all sets of virus-infected and -free isolates, and a phylogenetic tree was constructed based on the similarity of transcriptional pattern (Figure 3A). Transcriptomes of virus-free and -infected isolates in each pair were highly similar, whereas the isolates of different pairs showed a more diverse transcriptome. This view was also supported by the principal component analysis (PCA) data, which showed a close relationship between the virus-infected and -free isolates in each pair (Figure 3B).

Next, differentially expressed genes (DEGs) were extracted from the virus-infected and -free isolates in each set. The numbers of DEGs varied from 134 to 1507, in which IFM 65241 infected with the partitivirus showed the least and IFM 63847 co-infected with the partitivirus and polymycovirus showed the most DEGs (Figure 3C). From the DEG data, over- or underrepresented FunCat categories were explored in each set (See Table S3), which showed that metabolism was overrepresented in all strains. Then, we inspected gene expression levels in 59 SM clusters that have been estimated in *A. flavus*^29^^,^^30^^,^^31^^,^^32^ (See Table S4). In the 8 pairs of virus-infected and -free isolates, 26 to 37 SM clusters were not expressed regardless of virus infection. Altered gene expression was observed in one to six SM clusters in each strain, except for IFM 63449g (narnavirus). In IFM 61879 and IFM 49866 strains, the ustiloxin cluster was downregulated, and thus we attempted to detect ustiloxin production. Unexpectedly, these strains did not produce the compound under the tested conditions, regardless of virus infection (See Figure S11). Transcriptome data revealed that the ustYb gene, which is essential for ustiloxin production, was not expressed in IFM 61879 and IFM 49866 (See Figure S10 right), which might be associated with the lack of ustiloxin production.^33^

Virus infection is known to trigger RNA silencing in plants and fungi.^11^^,^^34^^,^^35^^,^^36^^,^^37^ Thus, we focused on virus effects on host genes related to RNA silencing. In *A. flavus*, genes involved in the RNA silencing system were not defined; thus, we searched for candidate genes by referring to the genes of *A. nidulans* and *A. fumigatus*.^35^^,^^38^ We found 4, 18, and 3 genes for Argonaute, Dicer, and RdRp functions. Unexpectedly, the expression levels for these genes were comparable between the virus-infected and -free isolates in all pairs (See Table S5).

### Comparison of DEGs across infected viruses

**Figure 4:**
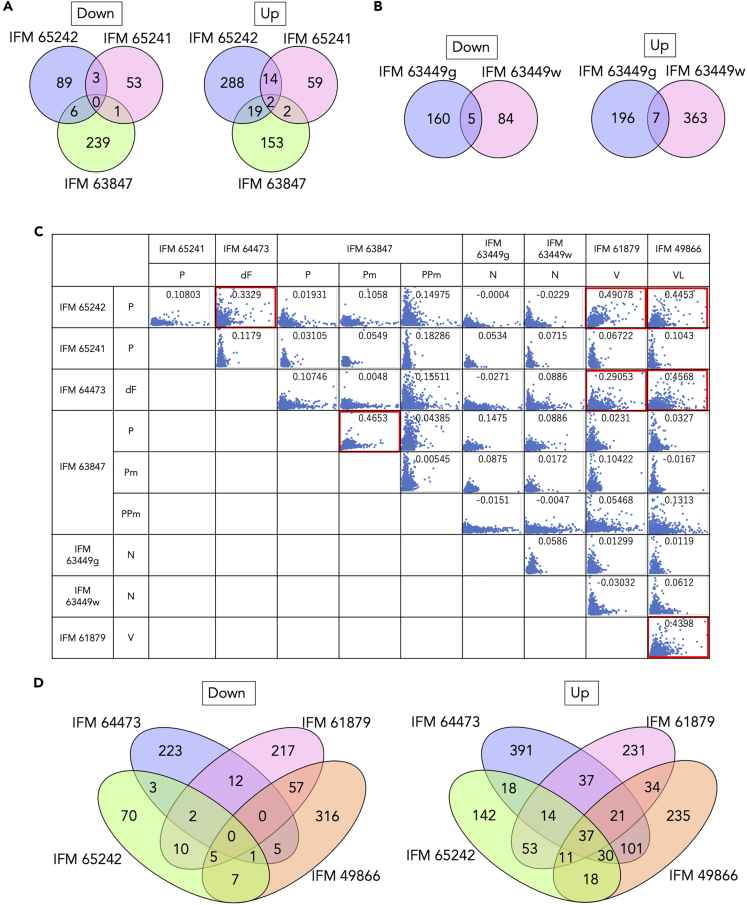
Comparison of DEGs across strains (A) Venn diagrams indicating the number of shared DEGs among strains infected with partitiviruses. (B) Venn diagrams indicating the number of shared DEGs among strains infected with narnaviruses. (C) Correlation coefficients for the expression ratio by virus infection across strains. Combinations that show a correlation value >0.25 are enclosed by a red line. Definitions of virus abbreviations are shown in Figure 1D. (D) Venn diagrams indicating the number of shared DEGs in IFM 65242 (partitivirus), IFM 64473 (deltaflexivirus), IFM 61879 (vivivirus), and IFM 49866 (virga-like virus); these showed a high correlation with each other in (C).

To gain a more comparative view of viral effects on host responses, DEGs were compared across the eight pairs. Although no DEGs were commonly included in all strains, several DEGs were shared in multiple strains. When compared among the 3 strains with partitivirus infection, only small proportions of the DEGs were common, indicating that a common transcriptional response to partitivirus infection was very limited (Figure 4A). In the same way, DEGs were compared between narnavirus-infected strains, IFM 63449g and IFM 63449w, which showed that five and seven genes were commonly down- and upregulated by viral infection, respectively (Figure 4B). These genes included NAD(P)H-dependent FMN reductase (AFLA_016350) and heat shock protein (AFLA_037820).

For a wider view of transcriptional responses to viral infection, correlation coefficients were calculated across the pairs based on the expression ratio of each gene (Figure 4C). Among the eight pairs of strains, relative similarity was observed among IFM 65242, IFM 64473, IFM 61879, and IFM 49866, despite differences in virus infection (Figure 4C). When DEGs were compared among these four pairs, more than half of the upregulated genes were shared with other strains in IFM 65242 and IFM 49866 (Figure 4D). Conversely, correlation coefficients were relatively low among the strains infected with the same species of partitivirus or narnavirus (Figure 4C), which was consistent with the small number of shared DEGs, as mentioned previously. These results suggest that host responses to virus infection are variable in each pair of virus-host strain.

### Virus transmission to different hosts

To further analyze virus-host specificity, we focused on the IFM 65242 and IFM 65241 strains that are infected with partitiviruses. There are three single nucleotide variations (SNVs) between genomes of the two partitiviruses (See Figure S12A), which may lead to different effects on host physiology. One SNV is located on the 3′-untranslated region of segment 4 (Base position: 1187), and the other two SNVs are inside of ORF1 (Base position: 369) or ORF8 (Base position: 690). While the SNV in ORF1 is synonymous, that in ORF8 results in amino acid change (A191T). To observe the effects of the SNVs, we generated the virus-free isolate with selection marker as a recipient. The marker-labeled IFM 65242 and IFM 65241 were represented as Ha (means host a) and Hb (means host b). Then, the virus was transmitted into the recipient isolates through hyphal fusion to obtain the fungal strains infected with original or different partitiviruses (See Figure S12B). First, the Ha and Hb isolates infected with the original partitivirus were obtained. Next, heterologous transmission was attempted, which resulted in IFM 65242 strain with the partitivirus derived from IFM 65241 and IFM 65241 strain with the partitivirus derived from IFM 65242. The partitivirus derived from IFM 65241 was named Va (means virus a) and the partitivirus derived from IFM 65242 was named Vb (means virus b). Virus transmission was confirmed by RT-PCR amplified from total RNA (See Figure S12C). We also confirmed that the titer of transmitted virus was largely comparable among the isolates, and all segments were transmitted as expected (See Figure S12D). Then, phenotype and secondary metabolism of these virus-swapped isolates were compared with the recipient (virus-free) isolates. Introduction of the viruses had no effects on colony appearance, colony growth rate, conidia production, mycelial morphology, aerial hyphae morphology, and secondary metabolism (See Figure S13).

### Transcriptome response to virus swapping

**Figure 5:**
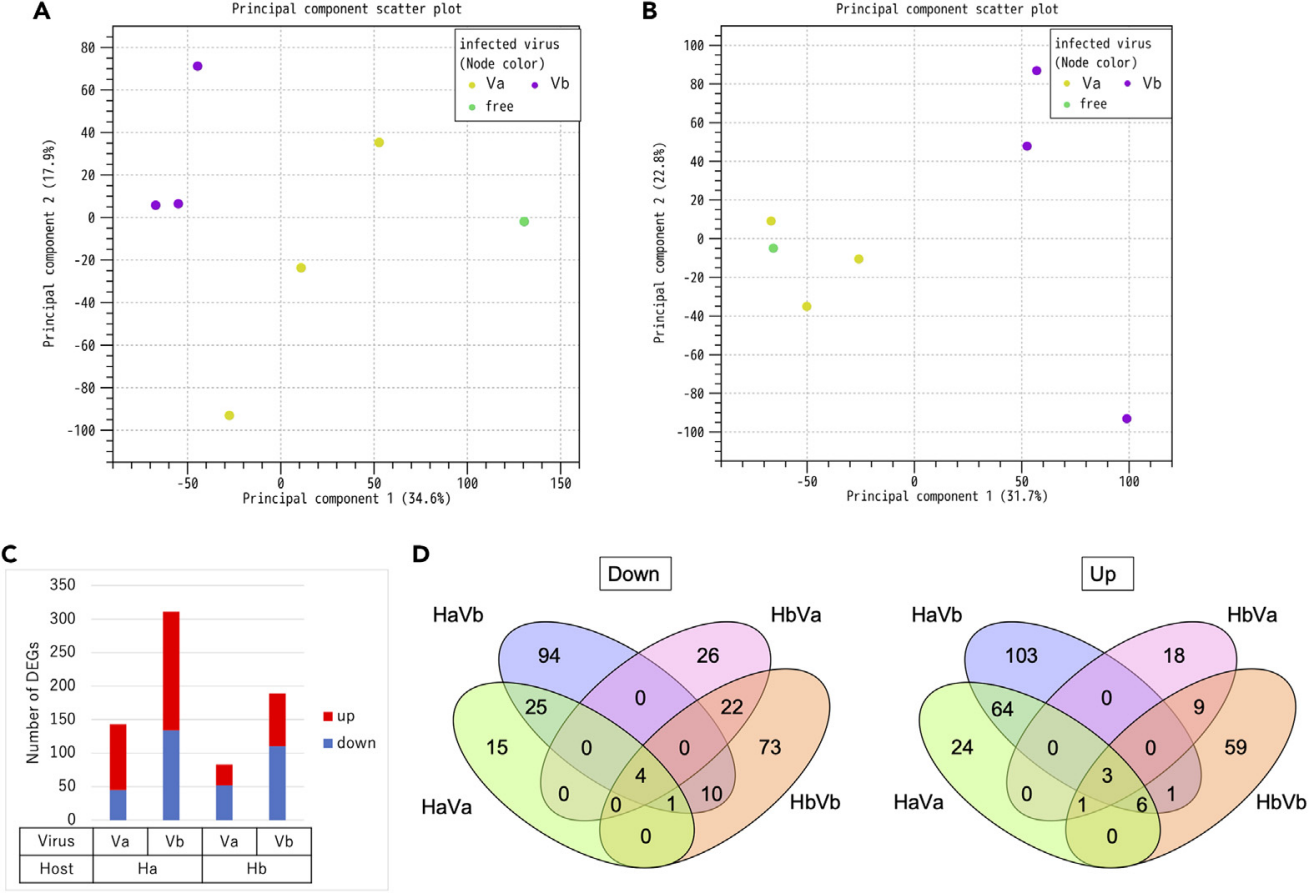
Comparative transcriptome analysis in virus-swapped isolates (A) PCA plot of gene expression in the Ha recipient isolate (free) and virus (partitivirus)-transmitted HaVa and HaVb isolates. (B) PCA plot of gene expression in the Hb recipient isolate (free) and virus (partitivirus)-transmitted HbVa and HbVb isolates. Recipient isolates (virus-free) are colored green and isolates harboring Va or Vb are presented in yellow or purple, respectively. (C) The number of DEGs affected by virus infection (compared with the corresponding recipient isolate). (D) Venn diagrams indicating the number of shared DEGs among the virus-swapped isolates. See also Tables S6 and S7.

For a deeper understanding, transcriptomes were compared among the recipient isolates (Ha and Hb), homo-transmitted isolates (HaVa and HbVb), and hetero-transmitted isolates (HaVb and HbVa). PCA analysis revealed that the hetero-transmitted isolates showed different gene expression pattern from the homo-transmitted isolates (Figures 5A and 5B). By comparing with the recipient isolate, DEGs were extracted for each isolate (Figure 5C). The number of DEGs for the hetero-transmitted isolates (HaVb) was larger than that for the re-introduced (homo-transmitted) isolates (HaVa). This result suggested that the original virus was more compatible with the fungal host than the virus from a different strain, even though the virus species is the same (different at 3 nt). In the case of Hb, however, the number of DEGs for the hetero-transmitted isolates (HbVa) was smaller than that for the homo-transmitted isolates (HbVb). Taken together, the data showed that Vb rather than Va had a larger effect on the transcriptional response of the host fungus.

When DEGs were compared between homo-transmitted and hetero-transmitted isolates, 67% (30/45 genes) of downregulated genes in HaVa were shared with HaVb, whereas 74% (73/98 genes) of upregulated genes were also shared (Figure 5D). In the case of Hb, 50% (26/52 genes) of downregulated genes in HbVa were shared with HbVb, whereas 42% (13/31 genes) of upregulated genes were also shared. The sets of such common DEGs (based on the same host) are shown in Tables S6 and S7, which included dynamin GTPase (AFLA_042270), P loop containing nucleoside triphosphate hydrolase (AFLA_042300), polyketide synthase (AFLA_118940), and O-methyltransferase (AFLA_119000). These results suggested that the same species but different strains of the mycovirus (different at three nucleotides) are able to affect the host transcriptome response in a similar manner.

Regarding the response of different fungal hosts to the identical virus, only 9% (4/45 genes) of downregulated genes in HaVa were shared with HbVa, whereas 4% (4/98 genes) of upregulated genes were also shared (Figure 5D). In the case of Vb, 11% (15/134 genes) of downregulated genes in HaVb were shared with HbVb, whereas 6% (10/177 genes) of upregulated genes were also shared. These data clearly showed that the transcriptome response of fungal strains to the same mycovirus is markedly different. Collectively, the DEGs and resultant cell physiology were largely unique to each combination of virus and host fungal genotypes.

## Discussion

In the present study, we identified multiple mycoviruses from environmental and clinical isolates of *A. flavus*, including a newly identified virus previously unassigned to a known virus species. Using these virus-infected strains, then we conducted a phenotypic study comparing virus-infected and -free isolates, and comparative transcriptome analysis to reveal virus effects. The results were then compared across the multiple virus-fungus combinations. Collectively, the different levels of comprehensive analyses highlighted a uniqueness of the viral effect that is highly dependent on both the viral and fungal strains. This is the first report to perform both phenotypic and transcriptomic analyses using multiple virus-fungus combinations, which helped to elucidate the range or magnitude of mycovirus effects on host fungi.

A survey of the virome of *A. flavus* isolates provided an interesting snapshot of the diversity of mycoviruses, and the FLDS method allowed us to identify several novel viral sequences from known mycovirus species. To determine if these novel sequences had been detected in the previous report, we retrieved the deposited metatranscriptome data, and the short reads were mapped to the novel sequences. As a result, these novel sequences were also present in the metatranscriptome data of the previous study.^28^ This indicates that conventional viral sequence detection methods, which detect viral sequences based on similarity to sequences in databases, have overlooked viral sequences that do not have distinctive annotations. In other words, only sequences that are highly conserved with other known viruses can in principle be found as viral sequences in conventional method. Similar cases have been reported previously.^39^

Comparisons of phenotypes between virus-infected and -free isolates have shown that mycoviruses are able to affect host physiology. While visible phenotypic alterations were limited, any variation was highly unique to the combination of virus and host strain in our set of strains. Indeed, different SM compounds were produced in a virus-dependent manner (Figure 2). Interestingly, there were no visible phenotypic alterations in any of the partitivirus-infected strains and narnavirus-infected strains compared with the virus-free isolates. These results suggested that the potential of such virus species to affect host physiology is very limited in *A. flavus*. On the other hand, partitiviruses and narnaviruses have been widely isolated from fungi such as *Aspergillus*, *Colletotrichum*, *Fusarium*, *Penicillium*, and *Saccharomyces*. Nerva et al.^40^ demonstrated that introduction of *Aspergillus ochraceus* virus, a partitivirus, into the virus-free *A. ochraceus* isolate resulted in overproduction of ochratoxin A. *Colletotrichum liriopes* partitivirus reduced the pathogenicity and conidia production of *C. liriopes*, which was confirmed through virus elimination and transfection experiments.^41^ *Fusarium equiseti* partitivirus 1 was also reported to confer hypovirulence and reduce the growth of the host fungus.^42^ Notably, AfPV1 has been isolated from an *A. flavus* isolate and was shown to influence colony morphology and conidia production of the host.^27^ This literature further supports that mycoviruses are able to affect host physiology in a manner dependent on the virus-fungus combination.

Several reports so far have shown viral effects on the host transcriptome.^25^^,^^26^^,^^43^^,^^44^^,^^45^ In most reports, however, single sets of virus-free and -infected isolates, but not multiple strain sets, were used. Thus, it has been difficult to appropriately evaluate the uniqueness of the viral effects between studies, even if the same fungal species were used. Hence, multiple sets of virus-free and -infected isolates are required for an overview of the level and range to which mycoviruses affect the host transcriptome. Thus, the results of our study are of great value for two reasons. First, our strain sets included one that showed no visible phenotypic alterations between the virus-free and -infected isolates. Indeed, partitiviruses and narnaviruses were shown to affect the host transcriptome, in which the expression of 134–495 genes was altered, even though no visible phenotypic changes were observed between the virus-free and -infected isolates. Therefore, our data clearly indicate that mycoviruses can affect host cells at the transcriptional level regardless of the phenotype.

Another contribution of this work is that a comparative view of fungal responses or adaptation to viral infection was provided using multiple strains. In particular, our data clearly indicated that RNA silencing was not activated in the *A. flavus* strains infected with viruses. This might be partly explained by the reports in which several mycoviruses act as RNA silencing suppressors.^46^^,^^47^ Taking into consideration the lack of effects on fungal growth, mycoviruses could behave as stealth symbionts in hosts. This view further suggests that a compatible relationship between mycovirus and host can be triggered not only by suppression of RNA silencing, which was established in the host, but also by viral adaptation, which could attenuate the viral effects so as not to lower the fitness of the fungal host. In this regard, satellite RNA was most recently demonstrated to attenuate the phenotypic and transcriptomic effects of Aspergillus flavus partitivirus 1 infection in *A. flavus*.^48^ Although the detailed molecular mechanism remains unknown, suppression machinery might also have been established in mycoviruses. To further understand the relationship between fungi and viruses, the mechanisms of such co-evolutionary relationships would be a focus in future studies.

The determinants of viral compatibility to its host and the ability to maintain infection are poorly understood. The frequency of virus elimination could be a clue to understanding how these relationships are established. Through a virus elimination experiment, we found that the partitivirus was more frequently removed by RBV treatment in IFM 63847 co-infected with partitivirus and polymycovirus compared with other partitivirus-infected strains. We also found that IFM 63847 with partitivirus infection showed greater effects on conidiation, aerial mycelia, conidial UV resistance, and SM production. From these results, we could conclude that there might be an association between virus effects on host physiology and virus compatibility with the host. This is a challenge for future work.

To evaluate the uniqueness of virus isolates in their roles, partitiviruses were swapped in identical natural host isolates. Our results indicated that there were no differences in phenotypic effects between the viral isolates; however, they showed differential effects in the transcriptomic response. Therefore, a change of only three nucleotides led to transcriptomic differences in the host. Our finding suggested that if mutations occur in the viral genome during infection, the host response to the virus may be accordingly changed at the transcriptional level. In this study, the virus transmission experiment using an identical fungal strain clearly showed the uniqueness of the viral isolate. Virus transmission is a powerful and promising tool to assess the virus-host relationship and to identify novel ways in which mycoviruses affect host cells.

### Limitations of the study

We have sequenced RNA viruses by FLDS method in this study. FLDS method is unable to capture DNA viruses and detect “extremely” low titer RNA viruses. Therefore, the possibility that DNA viruses and extremely low-titer RNA viruses remain infected in the “virus-free” isolates could not be ruled out.

Our results indicated that three SNVs in partitivirus genome differently affected host gene expression. However, the mechanism was remained to be resolved. This must be an open question for the next study.

RNA used for RNA sequencing was extracted from fungus cultured under the same condition as that used for secondary metabolite extraction (conidia were spread on YESA medium and cultured at 25°C for 7 days). Therefore, the conditions are different from that for morphological observation (spreading the conidia on PDA medium and incubation at 25°C for less than 5 days). Therefore, the expression data may not match the results of the morphological observation.

## STAR★Methods

### Key resources table

**Table:** 

REAGENT or RESOURCE	SOURCE	IDENTIFIER
**Biological samples**		

IFM 65242	Medical Mycology Research Center of Chiba University	IFM 65242
IFM 65241	Medical Mycology Research Center of Chiba University	IFM 65241
IFM 64473	Medical Mycology Research Center of Chiba University	IFM 64473
IFM 63847	Medical Mycology Research Center of Chiba University	IFM 63847
IFM 63449g	Medical Mycology Research Center of Chiba University	IFM 63449
IFM 63449w	Medical Mycology Research Center of Chiba University	
IFM 61879	Medical Mycology Research Center of Chiba University	IFM 61879
IFM 61226	Medical Mycology Research Center of Chiba University	IFM 61226
IFM 49866	Medical Mycology Research Center of Chiba University	IFM 49866

**Chemicals, peptides, and recombinant proteins**		

Ribavirin	Cayman Chemical	16757
2′-C-methyladenosine	Biosynth Ltd.	NM07917
7-deaza-2′-C-methyladenosine	Biosynth Ltd.	ND08351
2′-C-methylcytidine	Biosynth Ltd.	NM07918
pyrithiamine	Sigma-Aldrich	P0256
TRIzol reagent	Invitrogen	TRIzol reagent

**Critical commercial assays**		

EXPRESS One-Step Superscript qRT-PCR Kit	Invitrogen	1178101K
Nucleo Spin Plant II kit	MACHEREY-NAGEL	U0770A
NEBNext Ultra II FS DNA library prep kit for Illumina	New England Biolabs	E7805S
NEBNext Multiplex oligos for Illumina	New England Biolabs	E7335S
NEBNext Ultra II Directional RNA Library Prep Kit for Illumina	New England Biolabs	E7760S
NEBNext poly(A) mRNA Magnetic Isolation Module	New England Biolabs	E7490S

**Deposited data**		

Raw fastq data for host fungal genome	GenBank	PRJDB15839
Raw fastq data for viral genome	GenBank	PRJDB15327
Raw fastq data for transcriptome of virus-eliminated isolates	GenBank	PRJDB15326
Raw fastq data for transcriptome of virus swapped isolates	GenBank	PRJDB15621
Genome sequence of partitivirus in IFM 65242	GenBank	SAMD00585535
Genome sequence of partitivirus in IFM 65241	GenBank	SAMD00585536
Genome sequence of deltaflexivirus in IFM 64473	GenBank	SAMD00585537
Genome sequence of partitivirus in IFM 63847	GenBank	SAMD00585538
Genome sequence of polymycovirus in IFM 63847	GenBank	SAMD00585539
Genome sequence of narnavirus in IFM 63449g and IFM 63449w	GenBank	SAMD00585540
Genome sequence of vivivirus in IFM 61879	GenBank	SAMD00585541
Genome sequence of partitivirus in IFM 61226	GenBank	SAMD00585542
Genome sequence of virga like virus in IFM 49866	GenBank	SAMD00585543

**Recombinant DNA**		

pPTRI	Takara Bio Inc.	3621

**Software and algorithms**		

CLC genomics workbench software	QIAGEN N.V.	CLCGenomicsWorkbench 11.0.1
MEGA11	Tamura et al.^52^	https://www.megasoftware.net
trimAl	Capella-Gutiérrez et al.^53^	http://trimal.cgenomics.org/trimal
RAxML	Stamatakis^54^	https://cme.h-its.org/exelixis/software.html
FungiFun	Priebe et al.^55^	https://elbe.hki-jena.de/fungifun/

**Other**		

Poroshell120,EC-C18,3.0 × 100mm,2.7um	Agilent Technologies, Inc.	695975–302

### Resource availability

#### Lead contact

Further information should be directed to the lead contact, Daisuke Hagiwara (hagiwara.daisuke.gb@u.tsukuba.ac.jp).

#### Materials availability

The *Aspergillus flavus* strains used in this study were preserved in Medical Mycology Research Center of Chiba University. They have been collected through the National Bio-Resource Project (NBRP), Japan, and can be distributed. Accession numbers and source of isolates are listed in Table S1.

### Method details

#### Media

Fungi were cultivated on PDA medium (Difco, Becton Dickinson, NJ, USA) for growth and conidiation, YPDA medium [yeast extract (Difco, Becton Dickinson), peptone (Difco, Becton Dickinson), glucose (Nacalai Tesque, Japan), 1.5% agar (RIKAKEN Co., Ltd., Japan)] for colony growth without conidiation and establishing isolates derived from single colonies, or YESA medium [2% yeast extract, 15% sucrose (Nissin Sugar Manufacturing Co., Ltd., Japan), 1.5% agar] for extraction of chemical compounds and transcriptome analysis. IFM 63449g and IFM 63449w were isolated from the same sample and stocked in a single tube; however, the strains were distinguishable by phenotype and genotype.

#### Virus detection, definition, and elimination

We extracted dsRNA from mycelia cultured in liquid media and detected mycovirus-derived dsRNA by electrophoresis as previously reported.^49^ In brief, dsRNA was extracted using dsRNA extraction buffer [20 mM Tris–HCl, pH 6.8 (NIPPON GENE Co., Ltd., Japan), 200 mM NaCl (Nacalai Tesque), 2 mM EDTA (DOJINDO Co., Ltd, Japan), 1% SDS (Nacalai Tesque) and 0.1% (v/v) ·-mercaptoethanol (Nacalai Tesque)] and phenol chloroform (Nacalai Tesque), then purified using a cellulose resin (ADVANTEC Co., Ltd., Japan). The assembled virus sequence was analyzed by total RNA-seq based on terminal sequences as FLDS method ver. 3.^50^ The sequences were analyzed by blastx with e-value threshold of 1.0e-5. Open reading frames (ORFs) were screened by ORF finder, and ORFs more than 100 aa were shown and labeled as the results of conserved domain search in Figure 1. After confirmation of the sequence, we detected the virus using one-step RT-PCR with an EXPRESS One-Step Superscript qRT-PCR Kit, universal (Invitrogen, MA, USA), amplified from colony or total RNA. Primers used in this study are summarized in Table S8. We obtained some virus-free isolates using ribavirin (RBV; Cayman Chemical, MI, USA) treatment from each WT strain except for IFM 65242 and IFM 65241. Since partitiviruses in IFM 65242 and IFM 65241 strains were not removable by RBV treatment, 2′-C-methyladenosine (2CMA; Biosynth Ltd., UK), 7-deaza-2′-C-methyladenosine (7d2CMA; Biosynth Ltd.) and 2′-C-methylcytidine (2CMC; Biosynth Ltd.) were employed for IFM 65242 and IFM 65241 strains. We obtained no less than three independent virus free isolates, and three of them were used for phenotypic assay.

#### Morphology

For comparison of growth rate and observation of gross morphology, 10^2^ conidia were inoculated on the center of a PDA plate and incubated at 25°C. Colony diameter was measured every other day and growth rate (cm) per day was calculated. At 4 days after inoculation, the mycelia were cut with the agar medium attached and gross morphology was observed using a stereomicroscope. To compare conidia production, 10^3^ conidia were first spread on a PDA plate and incubated at 25°C for 4 days. The produced conidia were harvested from the colony surface using 3 mL of 0.05% Tween 20 (Nacalai Tesque) and the number of conidia were counted using a hemocytometer. For mycelial morphology, 10^2^ conidia were inoculated onto the center of PDA slide culture medium and incubated at 25°C for 3 days. After incubation, morphology was observed using an optical microscope. Minimum inhibitory concentrations (MICs) were determined according to the Clinical & Laboratory Standards Institute (CLSI) standard. For UV resistance, 10^2^ conidia were spread onto YPDA plates. The plates were UV irradiated (15 mJ/cm^2^, 253.7 nm: UV-C) in a UV-box (KENIS LTD., Japan). After treatment, the plates were immediately moved to an incubator and incubated at 30°C. After 1 day, the number of colonies appearing on the plate was counted using 3 replicates. The survival rate was calculated by comparing the colony number on the UV treated plates with that on the control plates without UV treatment. In the phenotypic assay, we used three biological replicates with three technical replicates for each. The data for representative biological replicate was presented.

#### Secondary metabolite (SM) analysis

Conidia (10^3^) were spread onto 5 mL of YESA slant medium in a 15-mL tube and incubated at 25°C for 7 days under aerobic conditions. Mycelia were freeze-dried along with the solid medium. To extract SMs, 10 mL of acetone (Nacalai Tesque) was added, and the suspension was sonicated for 10 min. A 5-mL aliquot of the acetone-extracted samples was dried *in vacuo*. After dissolving in methanol (Nacalai Tesque), the samples were passed through a C18 column (Cosmosil 140C_18_-OPN; Nacalai Tesque) and dried *in vacuo* again. The samples were dissolved in DMSO and analyzed using a 1260 Infinity LC system (Agilent Technologies, Inc., CA, USA) with a Poroshell 120 EC-C_18_ column (φ3.0 mm × 100 mm, particle size 2.7 μm; Agilent Technologies, Inc.). The high-performance liquid chromatography (HPLC) analytical condition was a gradient elution of 5–100% acetonitrile containing 0.5% acetic acid for 18 min at a flow rate of 0.8 mL/min. For detection of ustiloxin, 10^5^ conidia were incubated at 30°C in a mixture of 2.5 g cracked maize and 2.5 mL sterile water in a 50-mL tube for 7 days. A 10-mL aliquot of 70% (v/v) acetone was added to the tube, then the cracked maize and mycelia were removed. After vaporizing the acetone, 300 μL of the remaining water layer was mixed with an equal amount of ethyl acetate (Nacalai Tesque) for 1 h at room temperature. The water layer was analyzed using a 1260 Infinity LC system with a Poroshell 120 EC-C_18_ column. The HPLC analytical condition consisted of gradient elution of 2–30% acetonitrile containing 0.5% acetic acid for 20 min at a flow rate of 0.2 mL/min.

#### Whole genome sequencing

Conidia (10^3^) were inoculated in PDB liquid medium and cultured at 25°C for 4 to 7 days. Genomic DNA was extracted from the mycelia as previously reported.^51^ In brief, the mycelia were powdered in liquid nitrogen, from which genomic DNA was extracted with total nucleotide extraction buffer [200 mM NaCl, 20 mM Tris–HCl (pH 8.0), 2 mM EDTA (pH 8.0), 1% SDS, 0.1% 2-mercaptoethanol] and phenol-chloroform and then purified by ethanol precipitation and a Nucleo Spin Plant II kit (MACHEREY-NAGEL, Germany). DNA libraries for whole genome sequencing were generated using a NEBNext Ultra II FS DNA library prep kit for Illumina (New England Biolabs, MA, USA) and NEBNext Multiplex oligos for Illumina (Index primers Set 1) (New England Biolabs) according the manufacturer’s instructions.

The quality and concentration of libraries were examined using an Agilent 2100 bioanalyzer (Agilent Technologies, Inc.). Paired-end sequencing was performed by Novogene Co. (China). The data quality of genome sequencing is shown in Table S9. The raw read data was trimmed and assembled by CLC genomics workbench software (QIAGEN N.V., The Netherlands).

#### RNA-sequencing analysis

Conidia (10^3^) were spread on YESA plates and incubated at 25°C for 7 days. Total RNA was extracted from the mycelia using TRIzol reagent (Invitrogen) and a Pure Link RNA mini kit (Invitrogen), and then purified by ZYMO RNA clean & concentrator 5 (ZYMO RESEARCH, CA, USA) after DNaseI (Invitrogen) treatment. cDNA libraries for RNA-seq were generated by NEBNext poly(A) mRNA Magnetic Isolation Module (New England Biolabs), NEBNext Ultra II Directional RNA Library Prep Kit for Illumina (New England Biolabs), and NEBNext Multiplex Oligos for Illumina (Index Primer Set 1) (New England Biolabs) according to the manufacturer’s instructions.

The quality and concentration of libraries were confirmed using an Agilent 2100 bioanalyzer. Paired-end sequencing was performed by Novogene Co. (China). The data quality of RNA-sequencing is shown in Table S10. The raw read data was trimmed and mapped to the reference genome of *A. flavus* NRRL3357 using CLC genomics workbench software. The expression level for each gene was calculated as transcripts per million reads (TPM). The data were retrieved from two technical replicates for the representative isolate.

#### Phylogenetic analysis

To generate phylogenetic trees, MEGA11,^52^ trimAl,^53^ and RAxML^54^ were used. Bootstrap analysis was conducted with 1,000 samplings in RAxML.

#### Differential expression analysis and enrichment analysis

Differentially expressed genes (DEGs) between virus-infected and -free isolates were determined according to the following criteria: the genes were up- or down-regulated >2.5 times with a p value <0.05.

Functional enrichment analysis was performed using FungiFun,^55^ in which the DEGs were categorized^56^ and enriched categories were determined. The FunCat method is hierarchical in structure. All categories enriched in DEGs are presented in Table S3.

#### Virus transmission

For selection of recipient isolates with the transmitted virus, virus-free isolates were generated by transformation with *ptrA*, a selectable marker for pyrithiamine (Sigma-Aldrich Co. LLC, MO, USA). Transformation of *A. flavus* was performed using the protoplast-PEG method, as previously reported (Umemura et al., 2014). In brief, a plasmid carrying a pyrithiamine selectable marker, modified pPTRI (Takara Bio Inc., Japan), was introduced into protoplasts of *A. flavus* using PEG6000 (FUJIFILM Corporation, Japan). The pyrithiamine-resistant transformants were obtained after single spore isolation twice.

An outline of virus transmission is shown in Figure S12. The virus-free recipient isolate with selection marker and the virus-infected donor strain were co-inoculated on PDA plates and cultured at 25°C for 3 weeks. An agar block in the border region was excised, immersed in 0.05% Tween 20, and vortexed. The conidia suspension was spread onto Czapek Dox agar medium (Difco, Becton Dickinson) supplemented with pyrithiamine for selection. The pyrithiamine-resistant colonies were confirmed to harbor the virus by one-step RT-PCR, as described above. Virus-positive colonies for the 1st check were again single-colony isolated on Czapek Dox agar medium with pyrithiamine, and virus detection was performed. Then, the virus transmitted isolates were established, and the virus transmission rate was calculated from the number of virus-positive isolates divided by the number of first-screened isolates. We obtained three independent virus-transmitted isolates and analyzed morphology with three technical replicates for each biological replicate. For expression analysis, we used three virus-transmitted isolates of each pair and parental virus-free isolate in two technical replicates.

### Quantification and statistical analysis

p values were calculated using Student’s *t* test. p values less than 0.05 were considered to indicate a significant difference. ∗ means p value < 0.05, and ∗∗ means p value < 0.01.

## Data Availability

• Raw fastq data have been deposited at GenBank database repository and are publicly available as of the date of publication. • This paper does not report original code. • The sequences of viral genome were registered at GenBank. Accession numbers are listed in the key resources table.

## Supplemental information

Document S1. Figures S1–S13, Tables S1, and S6–S10

Table S2. The sequence of all segments of each virus detected in this study and the results of blastx, related to Figure 1

Table S3. List of categories enriched in DEGs by FunCat analysis, r elated to Figure 3

Table S4. Expression data of SM clusters and virus’s effect on its expression, related to Figure 3

Table S5. Fold change of gene expression related to RNA silencing, related to Figure 3

## References

[1] Tonka T., Walterová L., Čurn V. (2022). Biological control of pathogenic fungi: Can mycoviruses play an important role?. J. Cent. Eur. Agric..

[2] Nerva L., Turina M., Zanzotto A., Gardiman M., Gaiotti F., Gambino G., Chitarra W. (2019). Isolation, molecular characterization and virome analysis of culturable wood fungal endophytes in esca symptomatic and asymptomatic grapevine plants. Environ. Microbiol..

[3] Myers J.M., Bonds A.E., Clemons R.A., Thapa N.A., Simmons D.R., Carter-House D., Ortanez J., Liu P., Miralles-Durán A., Desirò A. (2020). Survey of early-diverging lineages of fungi reveals abundant and diverse mycoviruses. mBio.

[4] Khan H.A., Telengech P., Kondo H., Bhatti M.F., Suzuki N. (2022). Mycovirus hunting revealed the presence of diverse viruses in a single isolate of the phytopathogenic fungus *Diplodia seriata* from Pakistan. Front. Cell Infect..

[5] Villan Larios D.C., Diaz Reyes B.M., Pirovani C.P., Loguercio L.L., Santos V.C., Góes-Neto A., Fonseca P.L.C., Aguiar E.R.G.R. (2023). Exploring the Mycovirus Universe: Identification, Diversity, and Biotechnological Applications. J. Fungi.

[6] Dawe A.L., Nuss D.L. (2013). Hypovirus molecular biology: from Koch's postulates to host self-recognition genes that restrict virus transmission. Adv. Virus Res..

[7] Li H., Bian R., Liu Q., Yang L., Pang T., Salaipeth L., Andika I.B., Kondo H., Sun L. (2019). Identification of a novel hypovirulence-inducing hypovirus from Alternaria alternata. Front. Microbiol..

[8] Takahashi-Nakaguchi A., Shishido E., Yahara M., Urayama S.I., Sakai K., Chibana H., Kamei K., Moriyama H., Gonoi T. (2019). Analysis of an intrinsic mycovirus associated with reduced virulence of the human pathogenic fungus *Aspergillus fumigatus*. Front. Microbiol..

[9] Zhou L., Li X., Kotta-Loizou I., Dong K., Li S., Ni D., Hong N., Wang G., Xu W. (2021). A mycovirus modulates the endophytic and pathogenic traits of a plant associated fungus. ISME J..

[10] Lee K.M., Cho W.K., Yu J., Son M., Choi H., Min K., Lee Y.W., Kim K.H. (2014). A comparison of transcriptional patterns and mycological phenotypes following infection of *Fusarium graminearum* by four mycoviruses. PLoS One.

[11] Li K., Liu D., Pan X., Yan S., Song J., Liu D., Wang Z., Xie Y., Dai J., Liu J., Gao F. (2022). Deoxynivalenol Biosynthesis in *Fusarium pseudograminearum* Significantly Repressed by a Megabirnavirus. Toxins.

[12] Ninomiya A., Urayama S.I., Suo R., Itoi S., Fuji S.I., Moriyama H., Hagiwara D. (2020). Mycovirus-induced tenuazonic acid production in a rice blast fungus *Magnaporthe oryzae*. Front. Microbiol..

[13] Liu H., Wang H., Liao X.L., Gao B., Lu X., Sun D., Gong W., Zhong J., Zhu H., Pan X., Zhou Q. (2022). Mycoviral gene integration converts a plant pathogenic fungus into a biocontrol agent. Proc. Natl. Acad. Sci. USA.

[14] Chiba S., Salaipeth L., Lin Y.H., Sasaki A., Kanematsu S., Suzuki N. (2009). A novel bipartite double-stranded RNA mycovirus from the white root rot fungus *Rosellinia necatrix*: molecular and biological characterization, taxonomic considerations, and potential for biological control. J. Virol..

[15] Yu X., Li B., Fu Y., Jiang D., Ghabrial S.A., Li G., Peng Y., Xie J., Cheng J., Huang J., Yi X. (2010). A geminivirus-related DNA mycovirus that confers hypovirulence to a plant pathogenic fungus. Proc. Natl. Acad. Sci. USA.

[16] Yu X., Li B., Fu Y., Xie J., Cheng J., Ghabrial S.A., Li G., Yi X., Jiang D. (2013). Extracellular transmission of a DNA mycovirus and its use as a natural fungicide. Proc. Natl. Acad. Sci. USA.

[17] Zhang H., Xie J., Fu Y., Cheng J., Qu Z., Zhao Z., Cheng S., Chen T., Li B., Wang Q., Jiang D. (2020). A 2-kb mycovirus converts a pathogenic fungus into a beneficial endophyte for Brassica protection and yield enhancement. Mol. Plant.

[18] García-Pedrajas M.D., Cañizares M.C., Sarmiento-Villamil J.L., Jacquat A.G., Dambolena J.S. (2019). Mycoviruses in biological control: From basic research to field implementation. Phytopathology.

[19] van de Sande W.W.J., Vonk A.G. (2019). Mycovirus therapy for invasive pulmonary aspergillosis?. Med. Mycol..

[20] Rigling D., Robin C., Prospero S., Bamford D.H., Zuckerman M. (2021). Mycovirus-Mediated Biological Control” in Encyclopedia of Virology.

[21] Ghabrial S.A., Castón J.R., Jiang D., Nibert M.L., Suzuki N. (2015). 50-plus years of fungal viruses. Virology.

[22] Sato Y., Shahi S., Telengech P., Hisano S., Cornejo C., Rigling D., Kondo H., Suzuki N. (2022). A new tetra-segmented splipalmivirus with divided RdRP domains from *Cryphonectria naterciae*, a fungus found on chestnut and cork oak trees in Europe. Virus Res..

[23] Ikeda A., Chiba Y., Kuroki M., Urayama S.I., Hagiwara D. (2022). Efficient elimination of RNA mycoviruses in aspergillus species using RdRp-inhibitors ribavirin and 2’-C-methylribonucleoside derivatives. Front. Microbiol..

[24] Urayama S.I., Sakoda H., Takai R., Katoh Y., Minh Le T., Fukuhara T., Arie T., Teraoka T., Moriyama H. (2014). A dsRNA mycovirus, Magnaporthe oryzae chrysovirus 1-B, suppresses vegetative growth and development of the rice blast fungus. Virology.

[25] Wang L., Luo H., Hu W., Yang Y., Hong N., Wang G., Wang A., Wang L. (2018). De novo transcriptomic assembly and mRNA expression patterns of Botryosphaeria dothidea infection with mycoviruses chrysovirus 1 (BdCV1) and partitivirus 1 (BdPV1). Virol. J..

[26] Chun J., Ko Y.H., Kim D.H. (2020). Transcriptome analysis of *Cryphonectria parasitica* infected with Cryphonectria hypovirus 1 (CHV1) reveals distinct genes related to fungal metabolites, virulence, antiviral RNA-silencing, and their regulation. Front. Microbiol..

[27] Jiang Y., Wang J., Yang B., Wang Q., Zhou J., Yu W. (2019). Molecular characterization of a debilitation-associated partitivirus infecting the pathogenic fungus *Aspergillus flavus*. Front. Microbiol..

[28] Degola F., Spadola G., Forgia M., Turina M., Dramis L., Chitarra W., Nerva L. (2021). Aspergillus goes viral: ecological insights from the geographical distribution of the mycovirome within an *Aspergillus flavus* population and its possible correlation with aflatoxin biosynthesis. J. Fungi.

[29] Ye Y., Ozaki T., Umemura M., Liu C., Minami A., Oikawa H. (2018). Heterologous production of asperipin-2a: proposal for sequential oxidative macrocyclization by a fungi-specific DUF3328 oxidase. Org. Biomol. Chem..

[30] Uka V., Cary J.W., Lebar M.D., Puel O., De Saeger S., Diana Di Mavungu J. (2020). Chemical repertoire and biosynthetic machinery of the *Aspergillus flavus* secondary metabolome: A review. Compr. Rev. Food Sci. Food Saf..

[31] Costes L.H., Lippi Y., Naylies C., Jamin E.L., Genthon C., Bailly S., Oswald I.P., Bailly J.D., Puel O. (2021). The solvent dimethyl sulfoxide affects physiology, transcriptome and secondary metabolism of *Aspergillus flavus*. J. Fungi.

[32] Sweany R.R., Mack B.M., Moore G.G., Gilbert M.K., Cary J.W., Lebar M.D., Rajasekaran K., Damann K.E. (2021). Genetic responses and aflatoxin inhibition during co-culture of aflatoxigenic and non-aflatoxigenic *Aspergillus flavus*. Toxins.

[33] Umemura M., Nagano N., Koike H., Kawano J., Ishii T., Miyamura Y., Kikuchi M., Tamano K., Yu J., Shin-ya K. (2014). Characterization of the biosynthetic gene cluster for the ribosomally synthesized cyclic peptide ustiloxin B in *Aspergillus flavus*. Fungal Genet. Biol..

[34] Segers G.C., Zhang X., Deng F., Sun Q., Nuss D.L. (2007). Evidence that RNA silencing functions as an antiviral defense mechanism in fungi. Proc. Natl. Acad. Sci. USA.

[35] Özkan S., Mohorianu I., Xu P., Dalmay T., Coutts R.H. (2017). Profile and functional analysis of small RNAs derived from *Aspergillus fumigatus* infected with double-stranded RNA mycoviruses. BMC Genom..

[36] Mochama P., Jadhav P., Neupane A., Lee Marzano S.Y. (2018). Mycoviruses as triggers and targets of RNA silencing in white mold fungus *Sclerotinia sclerotiorum*. Viruses.

[37] Nguyen Q., Iritani A., Ohkita S., Vu B.V., Yokoya K., Matsubara A., Ikeda K.I., Suzuki N., Nakayashiki H. (2018). A fungal Argonaute interferes with RNA interference. Nucleic Acids Res..

[38] Hammond T.M., Bok J.W., Andrewski M.D., Reyes-Domínguez Y., Scazzocchio C., Keller N.P. (2008). RNA silencing gene truncation in the filamentous fungus *Aspergillus nidulans*. Eukaryot. Cell.

[39] Chiba Y., Oiki S., Yaguchi T., Urayama S.I., Hagiwara D. (2021). Discovery of divided RdRp sequences and a hitherto unknown genomic complexity in fungal viruses. Virus Evol..

[40] Nerva L., Chitarra W., Siciliano I., Gaiotti F., Ciuffo M., Forgia M., Varese G.C., Turina M. (2019). Mycoviruses mediate mycotoxin regulation in *Aspergillus ochraceus*. Environ. Microbiol..

[41] Zhu J.Z., Guo J., Hu Z., Zhang X.T., Li X.G., Zhong J. (2021). A novel partitivirus that confer hypovirulence to the plant pathogenic fungus *Colletotrichum liriopes*. Front. Microbiol..

[42] Mahillon M., Decroës A., Caulier S., Tiendrebeogo A., Legrève A., Bragard C. (2021). Genomic and biological characterization of a novel partitivirus infecting *Fusarium equiseti*. Virus Res..

[43] Li R., Zhou S., Li Y., Shen X., Wang Z., Chen B. (2018). Comparative methylome analysis reveals perturbation of host epigenome in chestnut blight fungus by a hypovirus. Front. Microbiol..

[44] Vainio E.J., Jurvansuu J., Hyder R., Kashif M., Piri T., Tuomivirta T., Poimala A., Xu P., Mäkelä S., Nitisa D. (2018). Heterobasidion partitivirus 13 mediates severe growth debilitation and major alterations in the gene expression of a fungal forest pathogen. J. Virol..

[45] Pedersen C.J., Marzano S.Y.L. (2022). Characterization of Transcriptional Responses to Genomovirus Infection of the White Mold Fungus, *Sclerotinia sclerotiorum*. Viruses.

[46] Yaegashi H., Yoshikawa N., Ito T., Kanematsu S. (2013). A mycoreovirus suppresses RNA silencing in the white root rot fungus, *Rosellinia necatrix*. Virology.

[47] Shimura H., Kim H., Matsuzawa A., Akino S., Masuta C. (2022). Coat protein of partitiviruses isolated from mycorrhizal fungi functions as an RNA silencing suppressor in plants and fungi. Sci. Rep..

[48] Jiang Y., Yang B., Liu X., Tian X., Wang Q., Wang B., Zhang Q., Yu W., Qi X., Jiang Y. (2022). A Satellite dsRNA Attenuates the Induction of Helper Virus-Mediated Symptoms in *Aspergillus flavus*. Front. Microbiol..

[49] Okada R., Kiyota E., Moriyama H., Fukuhara T., Natsuaki T. (2015). A simple and rapid method to purify viral dsRNA from plant and fungal tissue. J. Gen. Plant Pathol..

[50] Hirai M., Takaki Y., Kondo F., Horie M., Urayama S.I., Nunoura T. (2021). RNA viral metagenome analysis of subnanogram dsRNA using fragmented and primer ligated dsRNA sequencing (FLDS). Microbes Environ..

[51] Oiki S., Yaguchi T., Urayama S.I., Hagiwara D. (2022). Wide distribution of resistance to the fungicides fludioxonil and iprodione in Penicillium species. PLoS One.

[52] Tamura K., Stecher G., Kumar S. (2021). MEGA11: molecular evolutionary genetics analysis version 11. Mol. Biol. Evol..

[53] Capella-Gutiérrez S., Silla-Martínez J.M., Gabaldón T. (2009). trimAl: a tool for automated alignment trimming in large-scale phylogenetic analyses. Bioinformatics.

[54] Stamatakis A. (2014). RAxML version 8: a tool for phylogenetic analysis and post-analysis of large phylogenies. Bioinformatics.

[55] Priebe S., Kreisel C., Horn F., Guthke R., Linde J. (2015). FungiFun2: a comprehensive online resource for systematic analysis of gene lists from fungal species. Bioinformatics.

[56] Ruepp A., Zollner A., Maier D., Albermann K., Hani J., Mokrejs M., Tetko I., Güldener U., Mannhaupt G., Münsterkötter M. (2004). The FunCat, a functional annotation scheme for systematic classification of proteins from whole genomes. Nucleic Acids Res..

