# Supplementary material for: Experimental verification of strain-dependent relationship between mycovirus and its fungal host
Source: iScience. 2023 Jul 10;26(8):107337. doi: 10.1016/j.isci.2023.107337 (PMC10372822; doi:10.1016/j.isci.2023.107337)
Supplement: Document S1. Figures S1–S13, Tables S1, and S6–S10 [file mmc1.pdf]

iScience, Volume *26*

Supplemental information

Experimental verification of strain-dependent relationship between mycovirus and its fungal host

Misa Kuroki, Takashi Yaguchi, Syun-ichi Urayama, and Daisuke Hagiwara

Fig. S1 Detecting dsRNA virus by agarose gel electrophoresis, related to Table 1 and Figure 1

The dsRNA was extracted from cultured mycelium and analyzed by agarose gel electrophoresis. The gels were stained with GelRed and photographed by transilluminator and PrintGraph. P: Aspergillus flavus partitivirus1; Pm: Aspergillus flavus polymycovirus1; dF: Aspergillus flavus deltaflexivirus1; N: Aspergillus flavus narnavirus1; V: Aspergillus flavus vivivirus1; VL: Aspergillus flavus virga-like virus.

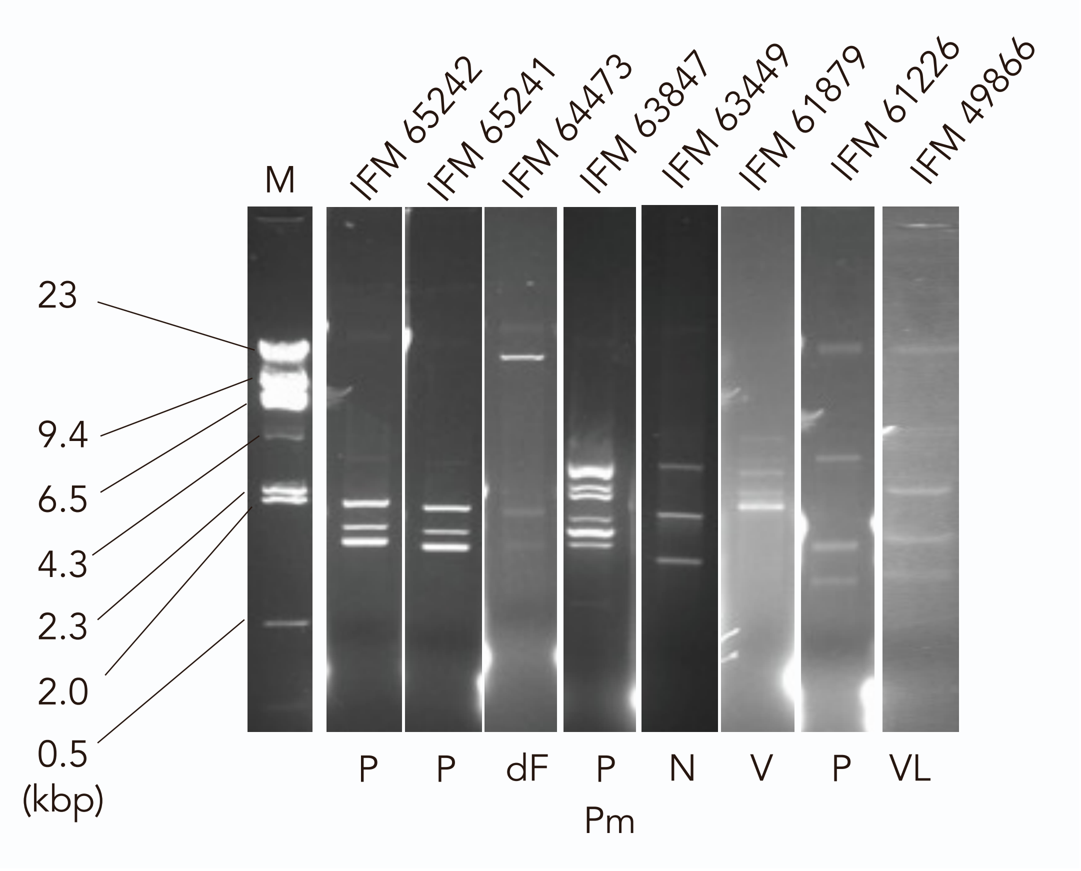

Fig. S2 Phylogenetic tree including identified partitivirus and polymicovirus, and the related viruses, related to Table 1 and Figure 1

Phylogenetic tree was constructed based on amino acid sequence of RdRp for the virus. The virus identified in this study was highlighted with a red rectangle.

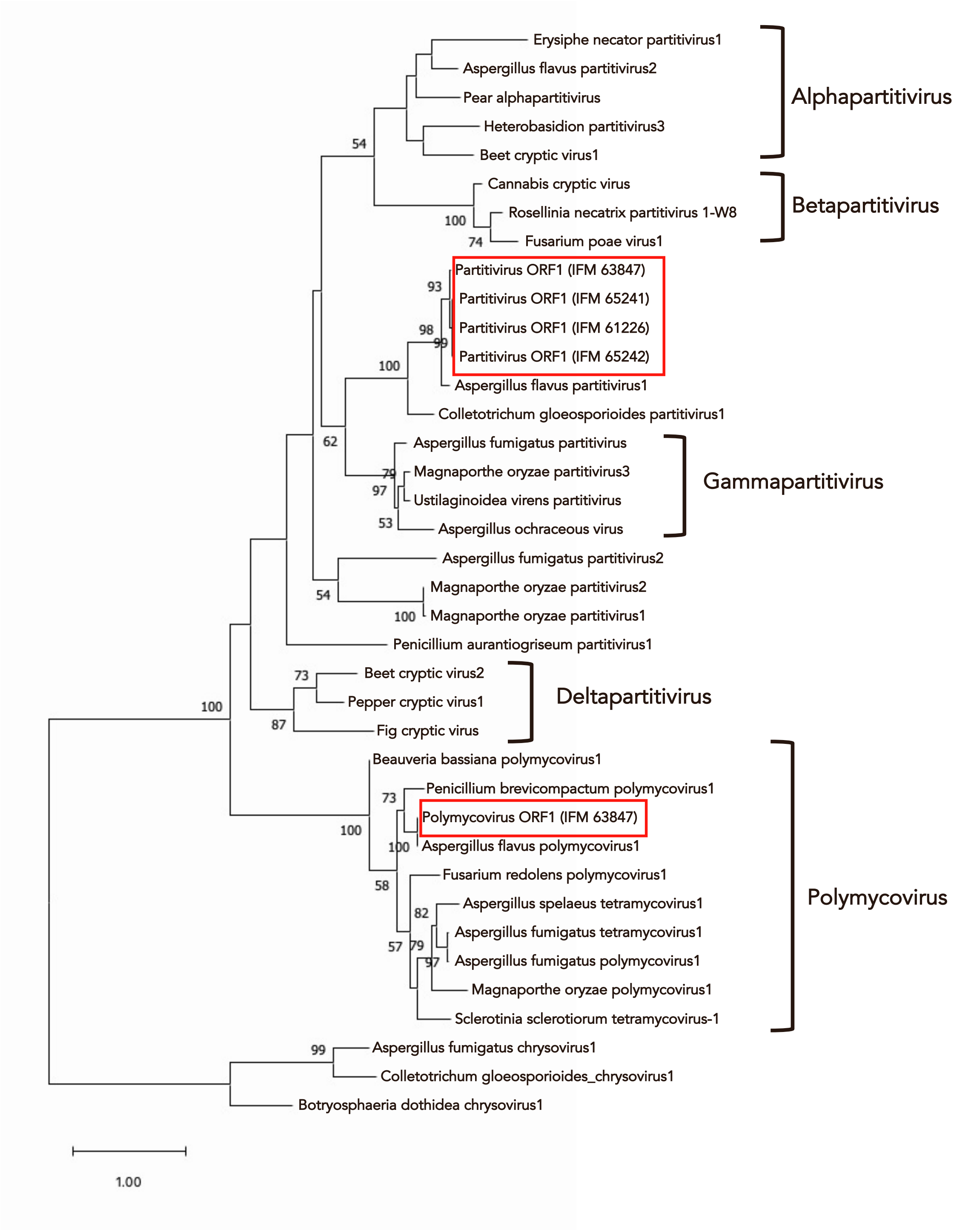

Fig. S3 Phylogenetic tree including identified deltaflexivirus (A) and narnavirus (B), and the related viruses, related to Table 1 and Figure 1

Phylogenetic tree was constructed based on amino acid sequence of RdRp for the virus. The virus identified in this study was highlighted with a red rectangle.

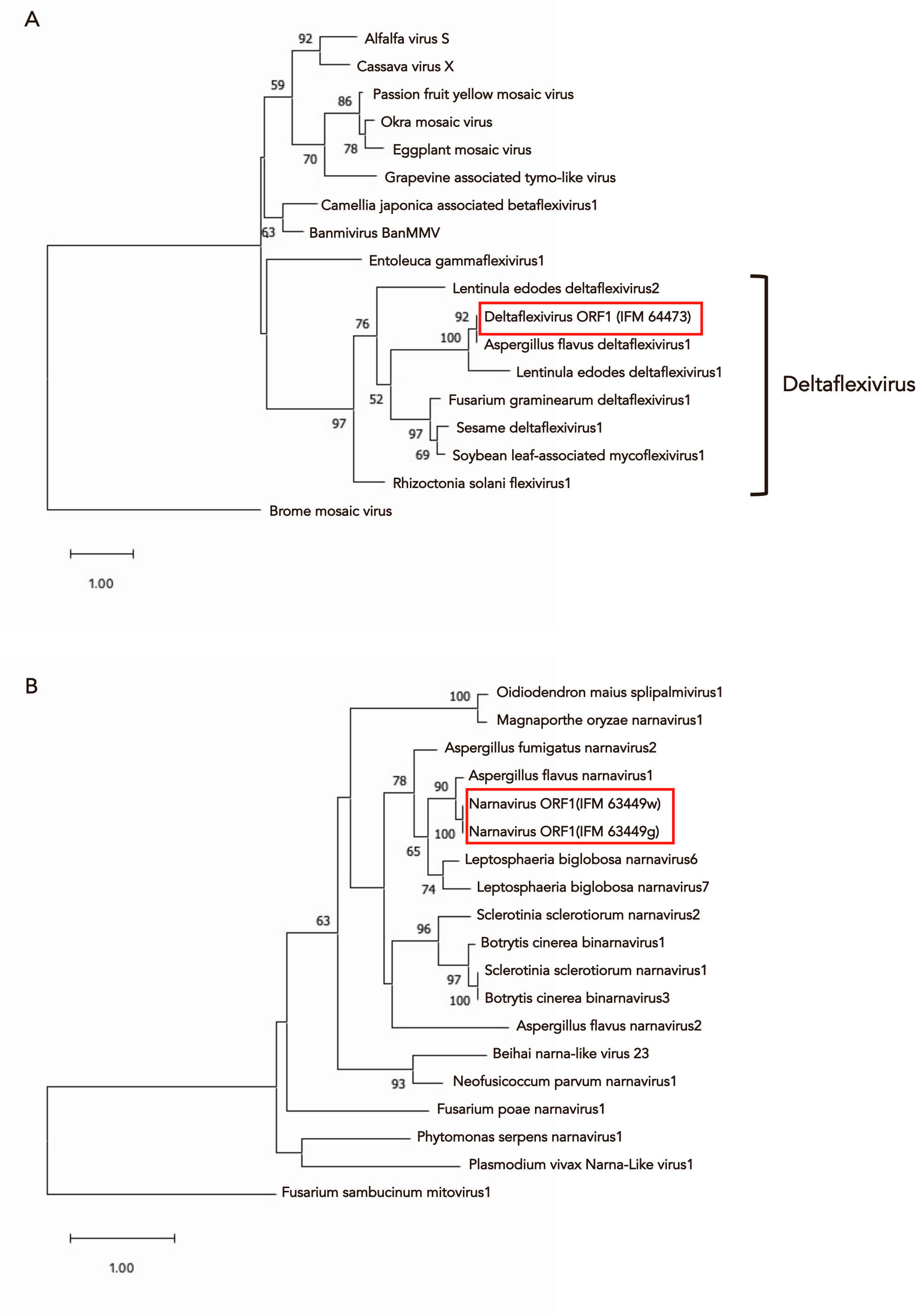

Fig. S4 Phylogenetic tree including identified vivivirus and virga like virus, and the related viruses, related to Table 1 and Figure 1

Phylogenetic tree was constructed based on amino acid sequence of RdRp for the virus. The virus identified in this study was highlighted with a red rectangle.

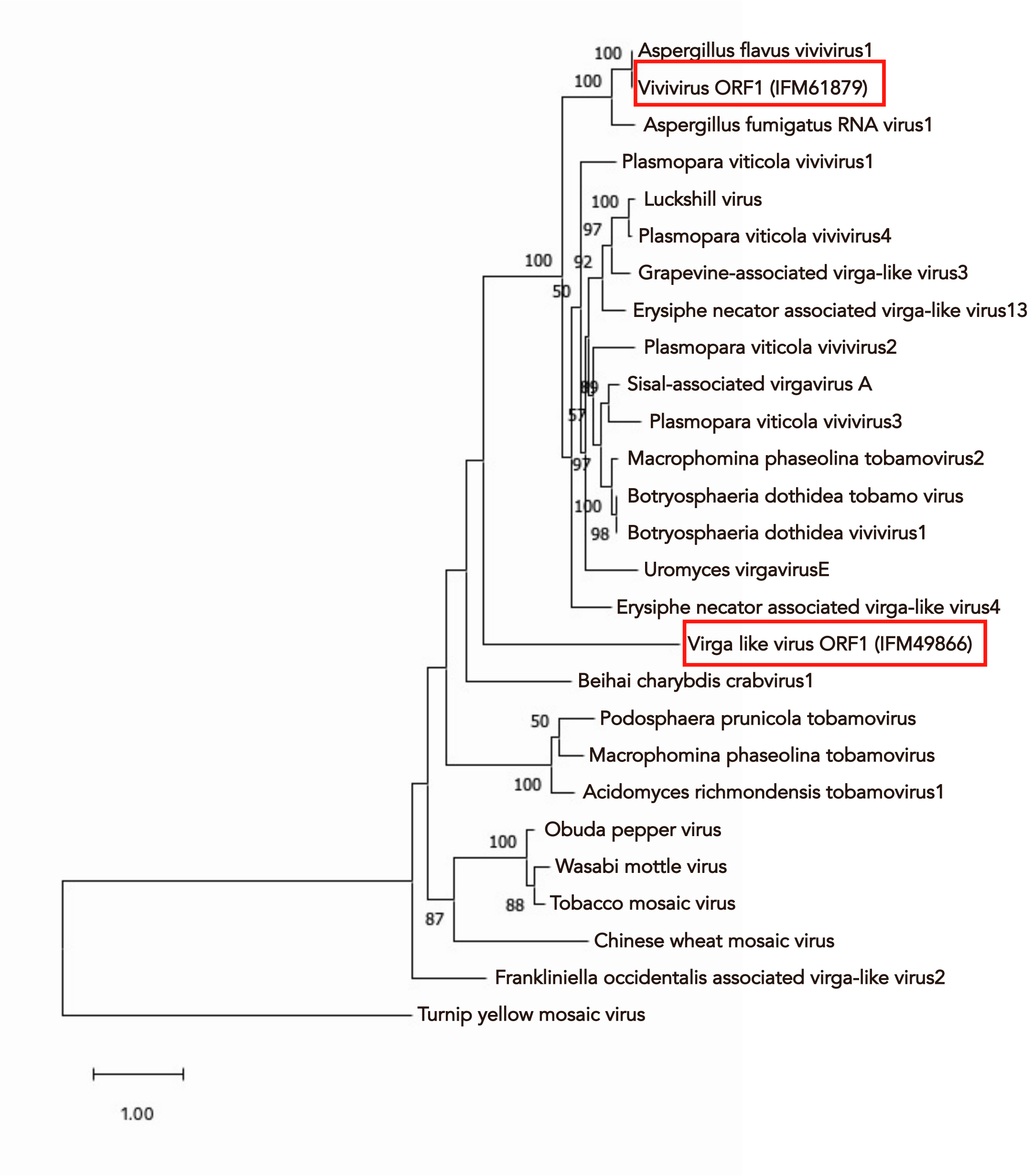

Fig. S5 Phylogenic tree of host fungi infected with mycoviruses, related to Table 1

The phylogenic tree was constructed based on the 1st chromosome by the Neighbor-joining method using CLC genomics workbench software. Definitions of virus abbreviations are shown in Fig. S1.

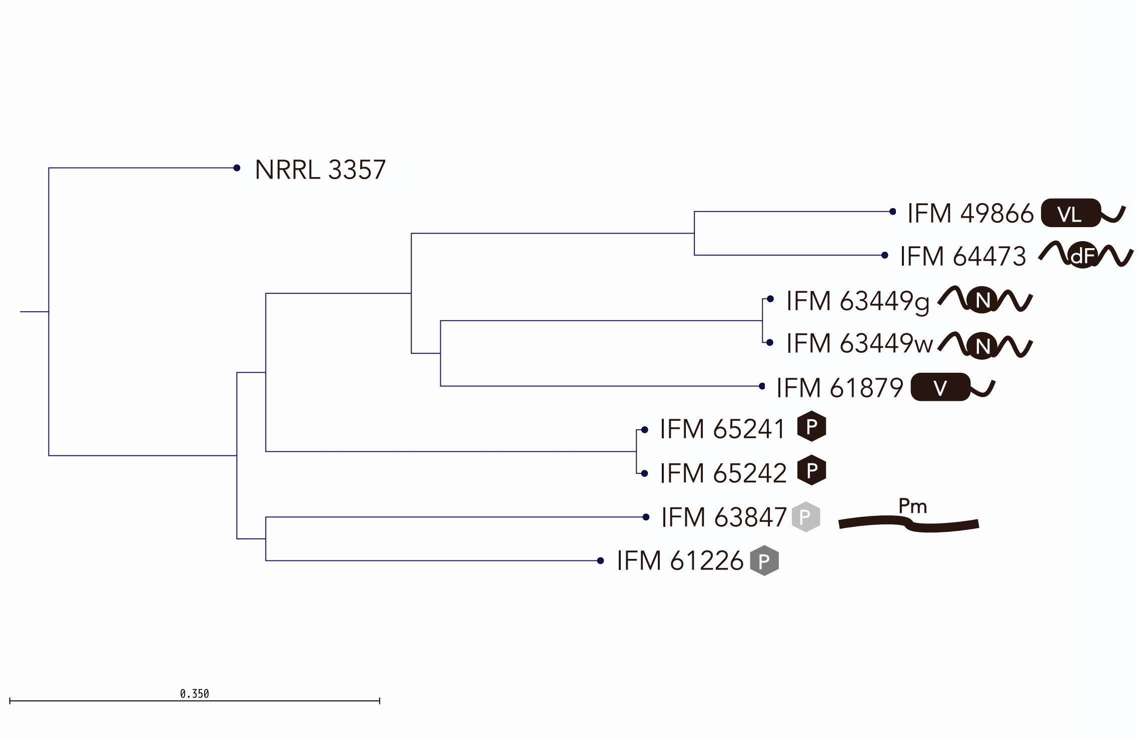

Fig. S6 Generation of virus-free isolates, related to "Virus detection, definition, and elimination" in STAR Method

(A) Frequency of virus-free colonies after virus elimination treatment. (B) Virus elimination was confirmed by RT-PCR. The lane indicated by "-"shows virus-eliminated isolates after treatment. Definitions of virus abbreviations are shown in Fig. S1.

A

Strain	Virus	RBV (0.01 mg/mL)	2CMA (0.2 mg/mL)	7d2CMA (0.2 mg/mL)	2CMC (0.2 mg/mL)
IFM 65242	P	0.0%	0.0%	0.0%	27.6%
IFM 65241	P	0.0%	0.0%	0.0%	34.8%
IFM 64473	dF	82.6%			
IFM 63847	P	9.1%			
	Pm	3.0%			
	P/Pm	15.2%			
IFM 63449g	N	1.7%			
IFM 63449w	N	2.7%			
IFM 61879	V	2.2%			
IFM 49866	VL	100.0%			

B

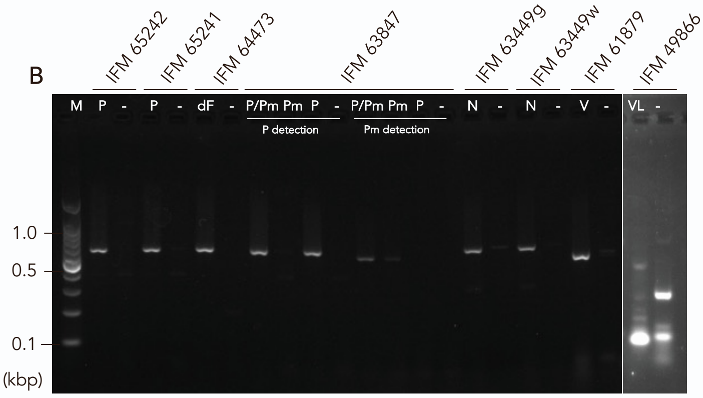

Fig. S7 Morphology of virus-infected wild-type strains and corresponding virus-free isolate, related to "Morphology" in STAR Mehod

(A) Conidia were inoculated on PDA in 6 cm petri dish and cultured at 25 °C for 4 days. (B) Gross morphology under stereomicroscopy was investigated using 4-day-old colonies. White bars indicate 1 mm. Definitions of virus abbreviations are shown in Fig. S1.

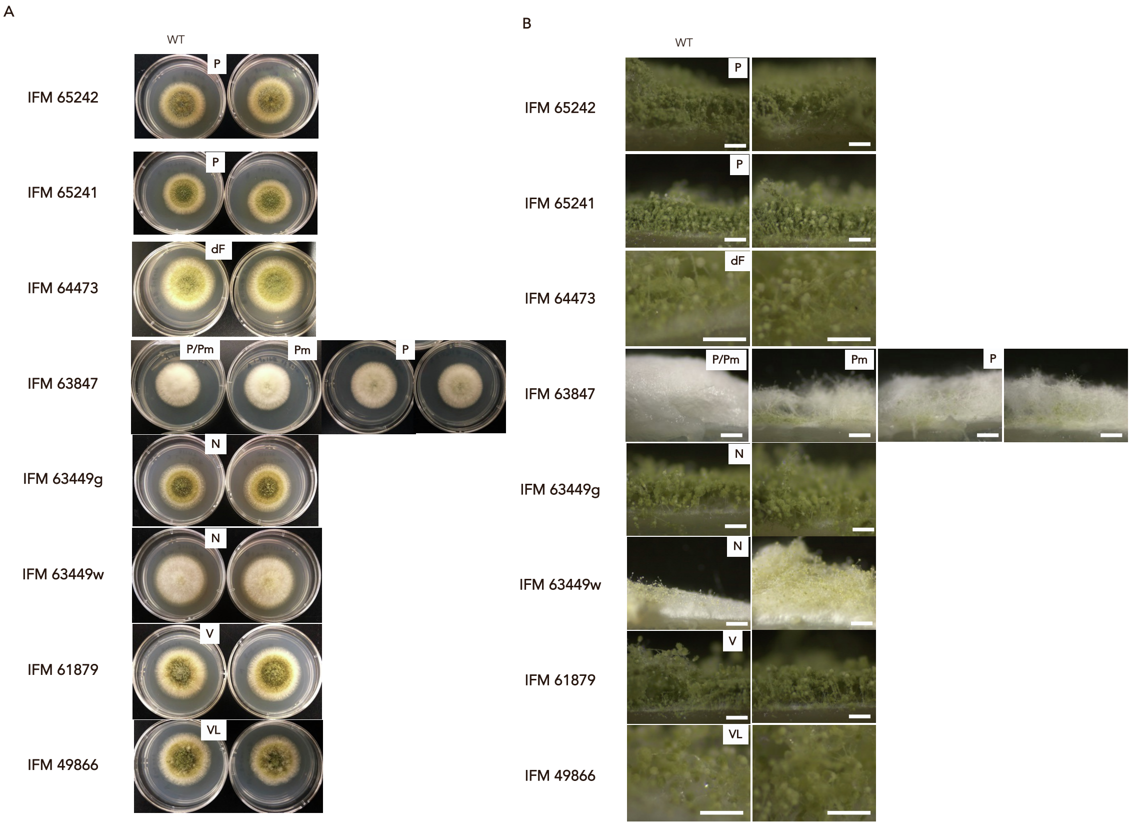

Fig. S8 Phenotypic characteristics of virus-infected wild-type strains and corresponding virus-free isolate, related to "Morphology" in STAR Mehod

(A) Number of conidia number per plate was counted using 4 day-old-colonies. The data was obtained using 3 replicates. (B) Resistance to UV irradiation was assessed in conidia spread on YPDA plates. Plates were compared with those without treatment, and the data was obtained using 3 replicates. (C) Antifungal sensitivity of the isolates. Definitions of virus abbreviations are shown in Fig. S1. Error bars indicate standard deviations. * means P value <0.05, and ** means P value <0.01 (Student's t-test.).

A

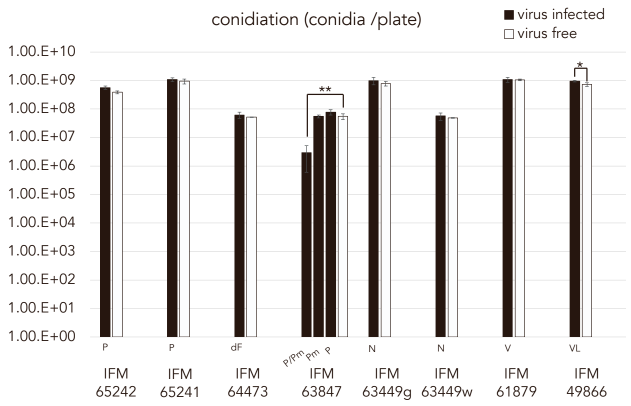

B

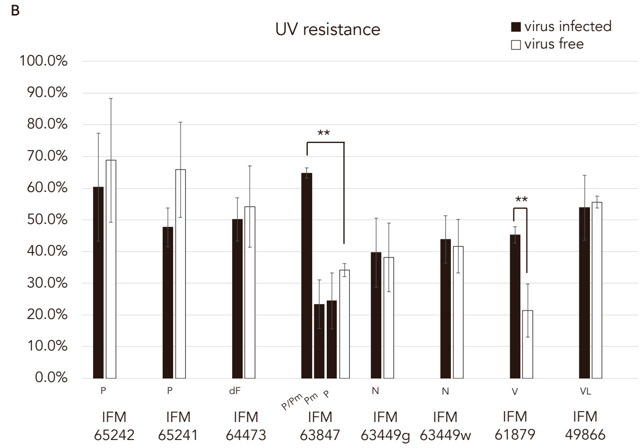

C

Strain	Virus	MIC (ug/mL)							
		MCFG	CPFG	AMPH	5-FC	FLCZ	ITCZ	VRCZ	MCZ
IFM 65242	P	≤0.015	0.12	2	>64	>64	0.25	1	2
	free	≤0.015	0.12	2	>64	>64	0.25	1	2
IFM 65241	P	≤0.015	0.12	2	>64	>64	0.25	1	1
	free	≤0.015	0.12	2	>64	>64	0.25	1	2
IFM 64473	dF	≤0.015	0.12	2	>64	>64	0.5	0.5	1
	free	≤0.015	0.12	2	>64	>64	0.5	0.5	1
IFM 63847	P/Pm	≤0.015	0.12	2	>64	>64	0.25	1	1
	Pm	≤0.015	0.12	1	>64	>64	0.25	0.5	1
	P	≤0.015	0.12	1	>64	>64	0.25	1	1
	free	≤0.015	0.12	1	>64	>64	0.25	1	2
IFM 63449g	N	≤0.015	0.12	2	>64	>64	0.25	0.5	1
	free	≤0.015	0.12	2	>64	>64	0.25	0.5	1
IFM 63449w	N	≤0.015	0.12	2	>64	>64	0.25	1	2
	free	≤0.015	0.12	2	>64	>64	0.25	1	2
IFM 61879	V	≤0.015	0.12	2	>64	64	0.25	0.5	1
	free	≤0.015	0.12	2	>64	>64	0.25	0.5	1
IFM 49866	VL	≤0.015	0.12	2	64	64	0.25	1	1
	free	≤0.015	0.12	2	>64	>64	0.25	1	1

Fig. S9 Production of cyclopiazonic acid (CPA) and aflatoxin B1(AFB1), related to Figure 2

Culture extracts were analyzed by HPLC to detect CPA and AFB1 at wavelengths of 214 nm and 330 nm, respectively. Metabolite production is indicated by an arrow over the peak. Definitions of virus abbreviations are shown in Fig. S1.

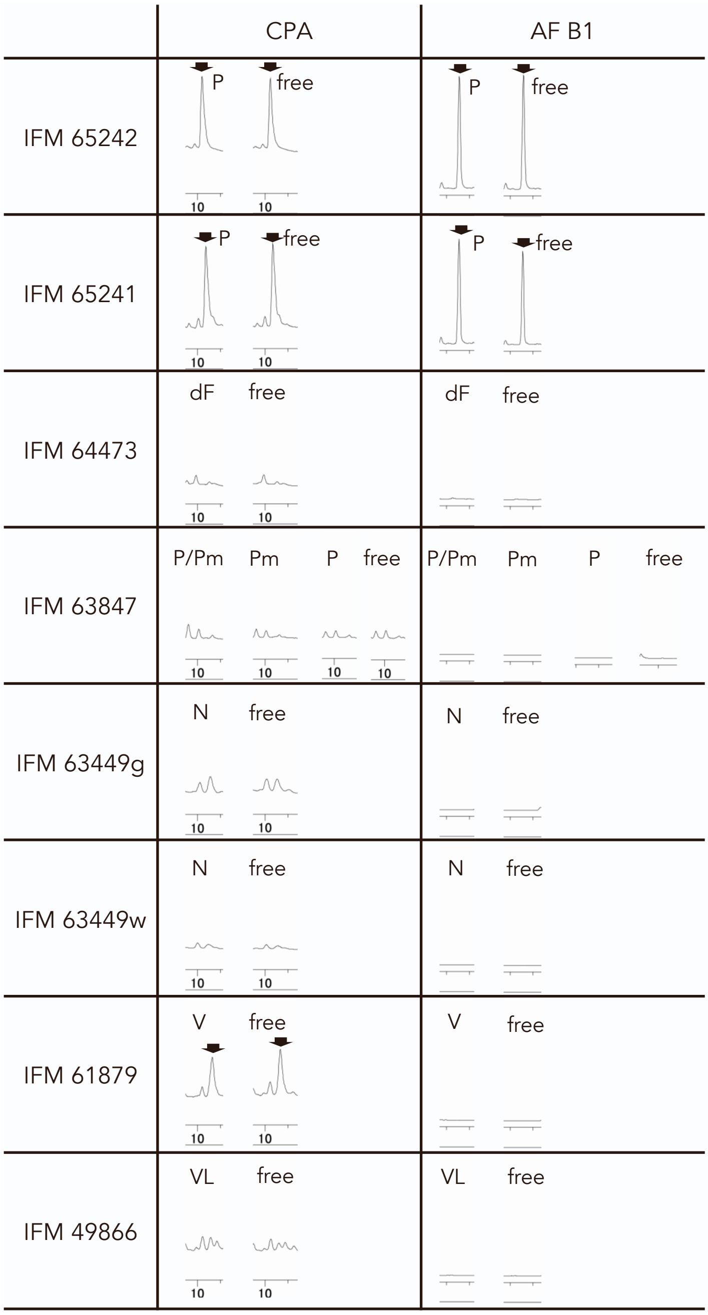

Fig. S10 Biosynthesis gene clusters for cyclopiazonic acid (CPA), aflatoxin (AF) and ustiloxin, related to Figure 3

Biosynthesis gene clusters for CPA, AF and ustiloxin cluster are shown. Arrows indicate the predicted ORFs. Genes containing long deletions or mutations that generate a stop codon were regarded as nonfunctional, and are thus not shown. According to the transcriptome analysis, gene expression level is indicated by color. Black: not expressed in either virus-infected or -free isolates; Red or Blue: up- or down-regulated in virus-infected strain compared with -free isolate, respectively. The other genes are shown in white.

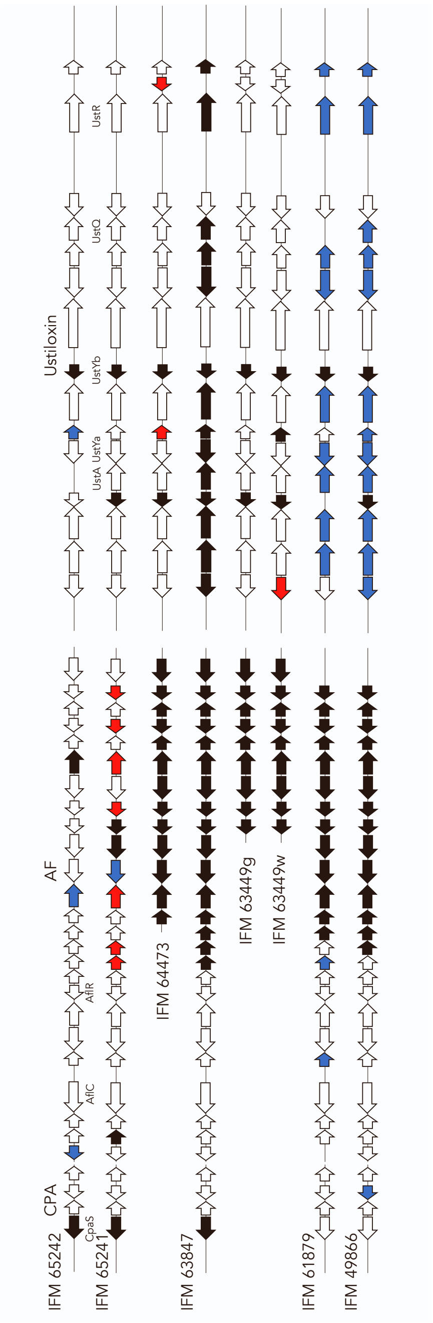

Fig. S11 Detection of ustiloxin production, related to Figure 3

Culture extracts were analyzed by HPLC at wavelength of 254 nm. No production of ustiloxin was detected.

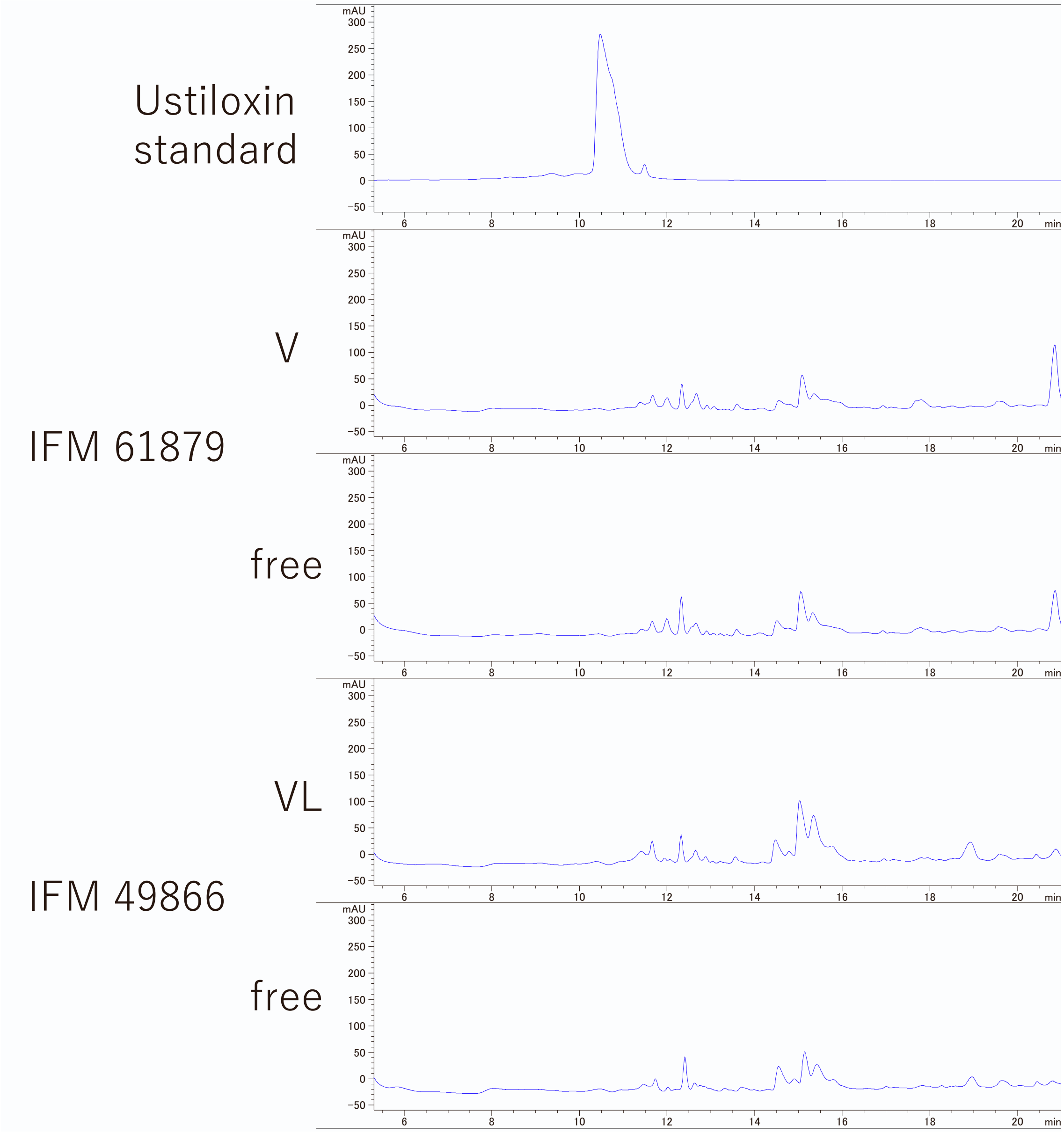

Fig. S12 Outline of virus transmission and confirmation by RT-PCR, related to "Virus transmission" in STAR Method

(A) Three single nucleotide variations between Va and Vb. Arrow heads indicate the position of difference and the adjacent tables show the nucleotide of Va or Vb in each point. (B) Schematic of the procedure for virus transmission and confirmation. (C) Virus transmission was confirmed by RT-PCR. The lane indicated by "-" shows the virus-free recipient isolate. (D) Virus titer and multipartitism in virus transmitted isolates were confirmed in dsRNA extraction or PCR amplified from cDNA transcribed from total RNA.

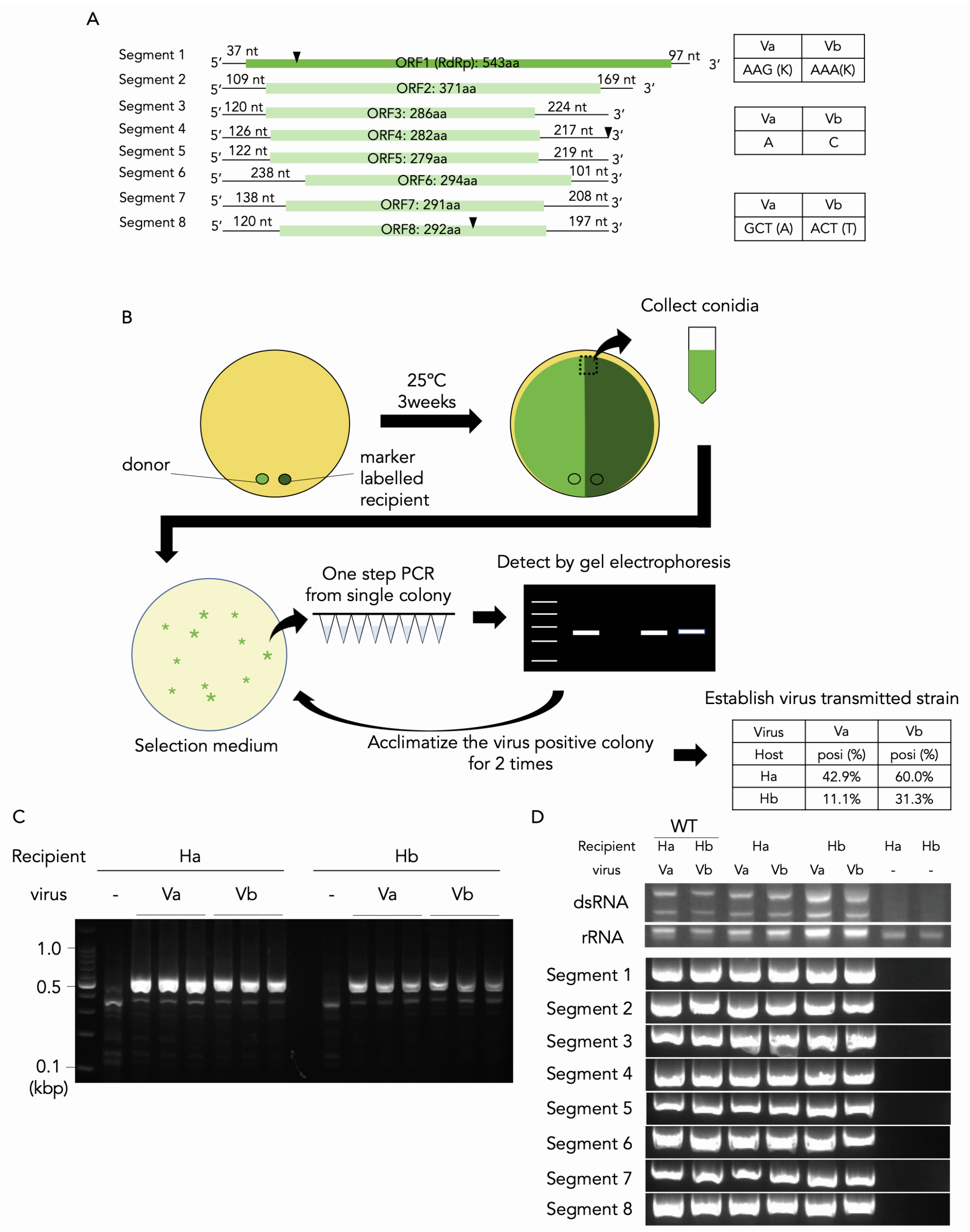

Fig. S13 Morphology of virus-transmitted isolates, related to Figure 5 and "Morphology" in STAR Method

(A) Colony appearance and aerial hyphae morphology of the virus-transmitted isolates. (B) Colony growth rate, conidia production, mycelial morphology, and secondary metabolism of the virus-transmitted isolates.

A

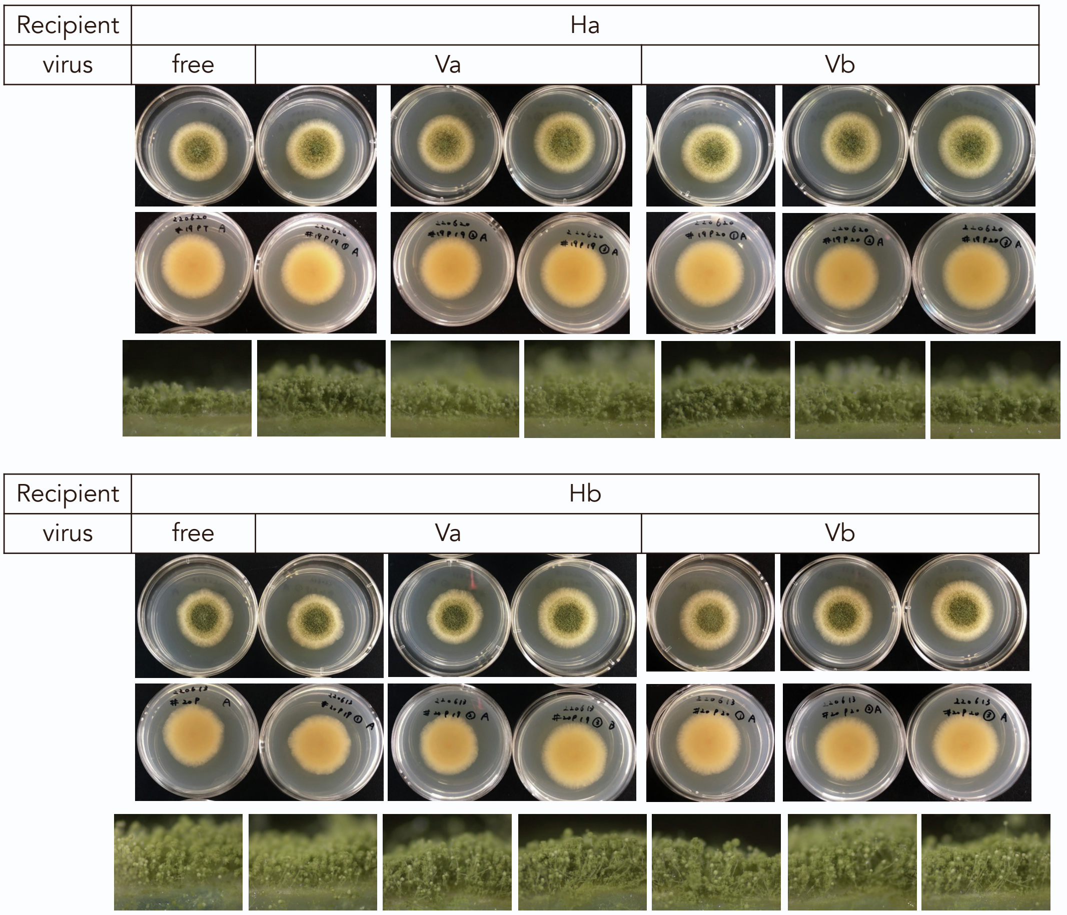

B

Recipient	Virus	Growth rate (cm/day)	Conidiation(conidia/plate)	Mycelial morphology	Cross section observation	HPLC peak
Ha	free	0.43 ± 0.01	1.07.E+09 ± 8.53.E+07			
	Va	0.44 ± 0.01	1.03.E+09 ± 1.43.E+08	not changed	not changed	not changed
		0.44 ± 0.00	1.09.E+09 ± 1.90.E+08			
		0.44 ± 0.01	9.10.E+08 ± 2.41.E+08			
	Vb	0.44 ± 0.01	1.09.E+09 ± 1.90.E+08	not changed	not changed	not changed
		0.43 ± 0.00	9.20.E+08 ± 2.43.E+08			
		0.45 ± 0.00	1.07.E+09 ± 2.36.E+08			
Hb	free	0.49 ± 0.01	1.17.E+09 ± 1.33.E+08			
	Va	0.48 ± 0.01	1.34.E+09 ± 6.06.E+07	not changed	not changed	not changed
		0.47 ± 0.02	1.33.E+09 ± 2.25.E+08			
		0.50 ± 0.03	1.38.E+09 ± 1.57.E+08			
	Vb	0.50 ± 0.00	1.41.E+09 ± 3.61.E+08	not changed	not changed	not changed
		0.50 ± 0.00	1.26.E+09 ± 3.64.E+08			
		0.51 ± 0.01	1.40.E+09 ± 3.44.E+08			

Table S1 List of Aspergillus flavus strains used in this study, related to "Materials availability" in STAR Method.

		Isolation source	Country	Another accession
IFM	52183	nursing items	Brazil	
IFM	52170	meeting room	Brazil	
IFM	52118	orpharyngeal swab	Brazil	
IFM	52117	orpharyngeal swab	Brazil	
IFM	52116	orpharyngeal swab	Brazil	
IFM	52115	orpharyngeal swab	Brazil	
IFM	49397	Arachis hypogaea,	Brazil	CBS 118.62, IFO 7600, RIB 1406, IMI 91548
IFM	49394	Arachis hypogaea,	Brazil	CBS 120.62
IFM	49393	Arachis hypogaea,	Brazil	CBS 119.62
IFM	49390	Arachis hypogaea,	Brazil	IFO 7600, CBS 118.62, IMI 91548, IFO 30106
IFM	57467	soil	China	
IFM	45909	chinese tea	China	
IFM	65979	sputum	Japan	
IFM	65920	ear	Japan	
IFM	65733	otorrhea (right)	Japan	
IFM	65712	tracheal suction phlegm	Japan	
IFM	65369	tissue from paranasal sinus	Japan	
IFM	65285	BALF	Japan	
IFM	65242	sputum, BALF	Japan	
IFM	65241	sputum, BALF	Japan	
IFM	65140	sputum	Japan	
IFM	65139	sputum	Japan	
IFM	65086	sputum	Japan	
IFM	65085	sputum	Japan	
IFM	65082	sputum	Japan	
IFM	65073	BALF	Japan	
IFM	64754	skin of scrotum	Japan	
IFM	64528	otorrhea	Japan	
IFM	64473	BALF	Japan	
IFM	64468	sputum	Japan	
IFM	63847	conjunctival sac	Japan	
IFM	63566	Lung	Japan	
IFM	63506	sputum	Japan	
IFM	63465	sputum	Japan	
IFM	63449g	inner of nail plate	Japan	
IFM	63449w	inner of nail plate	Japan	
IFM	63216	swab	Japan	
IFM	63215	swab	Japan	
IFM	63214	sputum	Japan	
IFM	62702	sputum	Japan	
IFM	62240	back (dead body)	Japan	
IFM	62162	bloody sputum	Japan	
IFM	61879	head (dead body)	Japan	
IFM	61854	rib (dead body)	Japan	
IFM	61850	pelvis (dead body)	Japan	

IFM	61527	right arm (dead body)	Japan	
IFM	61226	sputum	Japan	
IFM	61224	ear	Japan	
IFM	60655	sputum	Japan	
IFM	60519	sputum	Japan	
IFM	59975	otorrhea	Japan	
IFM	59894	peritoneal drainage	Japan	
IFM	58503	paranasal sinus biopsy	Japan	
IFM	57535	sputum	Japan	
IFM	55053	劇症型真菌性副鼻腔炎手術検体	Japan	
IFM	54693	歯肉	Japan	
IFM	54306	soil	Japan	
IFM	45911	soil	Japan	
IFM	63453	seaweed	Philippines	
IFM	49869	peanuts	Thailand	
IFM	49868	peanuts	Thailand	
IFM	49865	rice	Thailand	
IFM	49864	rice	Thailand	
IFM	49863	rice	Thailand	
IFM	49409	unknown	UK	
IFM	49405	unknown, Central Veterin. Labs.	UK	
IFM	60677	peanut cotyledons	USA	CBS 128202, ATCC 200026, NRRL 3357
IFM	55891	unknown	USA	NBRC 33021, ATCC 22546, IMI=CMI 370082
IFM	49401	soil	USA	NRRL 500, IFO 7540
IFM	49873	coffee bean	Vietnam	
IFM	49872	coffee	Vietnam	
IFM	49867	rice	Vietnam	
IFM	49866	rice	Vietnam	

Table S6 DEGs shared in Ha strain (IFM 65242), related to Figure 5.

Name		HaVa_Fold change	HaVb_Fold change	HbVa_Fold change	HbVb_Fold change
AFLA_009130	long-chain-fatty-acid CoA ligase	-2.94	-3.34		
AFLA_010360	quinone oxidoreductase	-3.02	-6.66	2.37	
AFLA_014500	plasma membrane channel protein (Aqy1)	-13.70	-16.11	-3.73	-4.90
AFLA_017640	C2H2 transcription factor (Rpn4)	-2.90	-5.15		
AFLA_024670		-2.99	-3.20		
AFLA_030780		-5.02	-2.62		
AFLA_038920	Gurmarin/antimicrobial peptide	-6.67	-11.27		
AFLA_042240		-2.72	-4.20	2.23	
AFLA_042270	dynamin GTPase	-992.10	-724.31	-15.43	-31.54
AFLA_042280		-5.13	-4.98		
AFLA_042290		-11.45	-11.27		
AFLA_042300	P-loop containing nucleoside triphosphate hydrolase	-1820.52	-561.89	-22.51	-89.98
AFLA_045960	DUF221 domain protein	-20.26	-14.45		
AFLA_053710	Cytochrome P450 superfamily	-10.31	-8.89	-1.85	
AFLA_053720	amino acid transporter	-40.34	-41.63		
AFLA_060780	hydrophobin	-46.97	-33.07	-3.19	-4.59
AFLA_062070		-4.33	-11.27		
AFLA_066440	S-adenosyl-L-methionine-dependent methyltransferase	-2.67	-5.46		-3.26
AFLA_066450	GTP cyclohydrolase I	-2.84	-7.32		
AFLA_068110	3-dehydroquinate dehydratase QutE	-2.54	-3.27		
AFLA_081900		-17.27	-30.64		
AFLA_083960		-11.45	-6.51		
AFLA_084840	Spartin-like	-35.79	-17.50		
AFLA_097370	Chitin synthesis regulation, Congo red resistance, RCR protein	-9.77	-15.25		
AFLA_099940		-5.25	-5.25		
AFLA_101840		-23.74	-11.03		
AFLA_106140		-5.32	-4.54		
AFLA_107650		-2.56	-4.39		
AFLA_119840		-2.74	-3.99	2.55	
AFLA_139200	aflQ/ ordA/ ord-1/ oxidoreductase/ cytochrome P450 monooxigenase	-2.99	-4.97	2.05	-1.71
AFLA_004030	NAD-dependent epimerase/dehydratase	4.66	5.20		
AFLA_004840	thioredoxin	3.84	4.55		
AFLA_005310	vacuolar ATP synthase proteolipid subunit	2.70	3.36		
AFLA_014030		7.57	10.53		
AFLA_014040		23.49	25.42	6.35	6.97
AFLA_014920	extracellular aspartic endopeptidase	2.87	4.82		
AFLA_016590		6.06	13.90	-4.94	-7.82
AFLA_016600	Oxoglutarate/iron-dependent dioxygenase	3.23	5.43	-2.76	-3.92
AFLA_017940	Armadillo-like helical	3.47	3.52	-2.21	
AFLA_018350		3.86	9.72	-3.55	-3.68
AFLA_020010		5.85	8.73		
AFLA_021020		3.09	4.04		
AFLA_022800	RTA-like protein	5.17	8.17		
AFLA_022830		2.81	7.78	-2.17	-2.49
AFLA_026790	fatty acid oxygenase PpoA	3.55	7.03	-2.32	-2.80
AFLA_029660	urea transporter (Dur3)	3.10	4.47		2.35
AFLA_031370		3.59	5.33		
AFLA_032010	FAD binding domain protein	3.88	5.78		
AFLA_033370	Cytochrome c oxidase biogenesis protein Cmc1-like	2.65	2.78		
AFLA_033890		2.81	3.43		
AFLA_035710	hscarg dehydrogenase	4.72	4.56	-4.09	-5.40
AFLA_038900	C6 finger domain protein	3.00	2.72		-2.81
AFLA_039590		2.88	5.03	-1.93	-2.95
AFLA_040220		4.97	6.03		
AFLA_046830	flotillin domain protein	4.20	4.82		2.00
AFLA_049770		3.44	4.73		

AFLA_050180	calcium transporter	2.81	4.02	-1.76	-1.82
AFLA_053410	Sodium/solute symporter superfamily	2.75	2.57		
AFLA_054490	neutral amino acid permease	4.32	6.48	-7.25	-2.50
AFLA_059290		2.62	5.54	-4.74	-9.08
AFLA_059790	alcohol dehydrogenase	4.41	4.84		
AFLA_060070		3.06	3.69		2.23
AFLA_060220	HotDog domain superfamily	7.85	5.47		
AFLA_060250	RNA polymerase II general transcription and DNA repair factor TFIIH	5.06	4.96		
AFLA_061460		2.57	2.61		
AFLA_063390	Peptidase	2.62	3.28		
AFLA_065610	Taurine dioxygenase TauD-like superfamily	2.56	3.33	1.61	2.61
AFLA_066050	Alpha-ketoglutarate-dependent dioxygenase AlkB-like superfamily	4.17	4.31		
AFLA_074940	viral-enhancing factor	2.53	5.51	-2.31	-2.10
AFLA_075840	hyaluronan synthase	2.84	3.35	1.86	2.74
AFLA_079590		2.99	2.57		
AFLA_082200	AAA family ATPase	2.80	2.80	1.78	2.73
AFLA_085540		2.97	5.83	-1.59	
AFLA_085820	Glycoside hydrolase family	4.26	5.35		
AFLA_089430	Transcription factor domain	5.92	8.46		
AFLA_095420	small oligopeptide transporter, OPT family	3.76	3.72		2.23
AFLA_097830	xylulose kinase	2.74	2.91		
AFLA_099550		3.31	7.32		
AFLA_099740		2.68	3.43		
AFLA_099820	Zinc finger, FYVE/PHD-type	7.79	9.72		2.30
AFLA_102920		3.14	3.01		
AFLA_108070	Fluoroacetyl-CoA thioesterase	4.17	4.48		
AFLA_110730		2.72	4.79		
AFLA_111670	Complex 1 LYR protein domain	2.51	2.62		
AFLA_111860	Lysine methyltransferase	6.50	9.12		
AFLA_113290		2.84	2.88		
AFLA_117080	iron-regulated transporter	3.32	3.88		
AFLA_118600		4.55	3.82		
AFLA_118940	polyketide synthase	8.27	9.74	9.94	9.54
AFLA_118950	Alpha/Beta hydrolase fold	6.17	6.71		12.40
AFLA_118970	MAK1-like monooxygenase	4.01	3.83	2.32	2.58
AFLA_118980	mitochondrial carrier protein Leu5	2.72	2.76	1.88	2.19
AFLA_119000	O-methyltransferase	18.39	19.83	26.08	25.54
AFLA_121580	C-4 methyl sterol oxidase	2.81	3.82		
AFLA_121760	DSBA-like thioredoxin domain protein	2.98	2.97		
AFLA_125580	FAD-binding, type PCMH-like superfamily	3.67	3.46		
AFLA_126500	Zn(2)-C6 fungal-type DNA-binding domain superfamily	2.63	3.10	2.12	2.21
AFLA_126530	Taurine dioxygenase TauD-like superfamily	4.42	8.16	-3.21	-4.36
AFLA_126550	ornithine decarboxylase	3.06	5.15		
AFLA_131460	hydrophobin	12.39	14.91		3.09
AFLA_137920	MFS siderophore iron transporter	3.05	4.00		
AFLA_138680	pentachlorophenol 4-monooxygenase	3.35	3.21		
AFLA_138700		3.67	7.82		

Table S7 DEGs shared in Hb strain (IFM 65241), related to Figure 5.

Name		HaVa_Fold change	HaVb_Fold change	HbVa_Fold change	HbVb_Fold change
AFLA_003750				-13.24	-52.92
AFLA_012060	choline oxidase (CodA)	1.42	1.85	-2.58	-6.75
AFLA_016590		6.06	13.90	-4.94	-7.82
AFLA_016600	Isopenicillin N synthase-like superfamily	3.23	5.43	-2.76	-3.92
AFLA_018350		3.86	9.72	-3.55	-3.68
AFLA_022820		2.32	6.98	-2.68	-3.88
AFLA_034570				-36.52	-38.09
AFLA_035710	hscarg dehydrogenase	4.72	4.56	-4.09	-5.40
AFLA_042270	dynamin GTPase	-992.10	-724.31	-15.43	-31.54
AFLA_042300	P-loop containing nucleoside triphosphate hydrolase	-1820.52	-561.89	-22.51	-89.98
AFLA_046170	Protein-tyrosine phosphatase-like			-15.09	-9.68
AFLA_051590	dihydrofolate synthase/folylpolyglutamate synthase	1.72	2.79	-2.79	-6.05
AFLA_053710	Cytochrome P450 superfamily	-46.97	-33.07	-3.19	-4.59
AFLA_057600	heat shock protein	1.77	2.08	-3.38	-6.37
AFLA_059290		2.62	5.54	-4.74	-9.08
AFLA_060780	hydrophobin	-13.70	-16.11	-3.73	-4.90
AFLA_061000				-9.75	-67.74
AFLA_069490				-7.57	-2.63
AFLA_077640	Six-hairpin glycosidase superfamily			-5.69	-4.11
AFLA_095460		1.94	2.63	-3.77	-6.20
AFLA_113350		1.90	2.55	-5.15	-24.63
AFLA_126150	Nucleotidyltransferase superfamily	1.77	1.95	-3.63	-5.06
AFLA_126530	Taurine dioxygenase TauD-like superfamily	4.42	8.16	-3.21	-4.36
AFLA_126750	extracellular glycine-rich protein			-7.00	-6.39
AFLA_131900				-5.58	-7.45
AFLA_137640	Velvet domain superfamily			-3.07	-20.06
AFLA_014040		23.49	25.42	6.35	6.97
AFLA_018470				8.52	8.17
AFLA_041310	oxidoreductase		-2.58	3.33	3.08
AFLA_065140	Bacterial exopeptidase dimerisation domain			3.72	7.38
AFLA_073360				12.38	15.40
AFLA_077630				2.70	2.69
AFLA_078140	acetyltransferase, GNAT family			3.42	5.86
AFLA_101470	glyceraldehyde-3-phosphate dehydrogenase	8.12		8.87	8.59
AFLA_105630	cytochrome P450 monooxygenase	-1.58	-2.05	2.66	2.53
AFLA_118940	polyketide synthase	8.27	9.74	9.94	9.54
AFLA_119000	O-methyltransferase	18.39	19.83	26.08	25.54
AFLA_131480				5.13	6.49
AFLA_139230	aflI/ avfA/ cytochrome P450 monooxygenase		-3.08	4.84	3.87

Table S8 Sequence of primers used in this study, related to "Virus detection, definition, and elimination" in STAR Method.

Name	Sequence 5' -> 3'	Purpose	Product size
Af_P-F	CCTTGGACAAAGCCGTTTCG	Detecting partitivirus	558 bp
Af_P-R	AACAGCAGCAGGGTACATCC		
Af_Df-F	TACGAGACTGTACAGGGCGA	Detecting deltaflexivirus	561bp
Af_Df-R	GTGCACGTGTGGGTTAACAC		
Af_Pm-F	GGCTTGGTAGTGACACGTCA	Detecting polymycovirus	517 bp
Af_Pm-R	GGCGCGTCACATCAATGTAC		
Af_N-F	CCGGAAGCCATGTCTGAAGT	Detecting narnavirus	579 bp
Af_N-R	AGGAGGTGGGCTCACTGTAT		
Af_V-F	ACGCCTGACAAGAGACTGTG	Detecting vivivirus	512bp
Af_V-R	CCGGCCATTCGAGAGTAGAC		
Af_VL-F	ACACCGGAGAAGGTTAAGCG	Detecting virga like virus	509bp
Af_VL-R	AGATATGCCTTCACACCGGC		
Af_PV_seg2-F	TGACTCGCCTGAACCTGAAC	Detecting partitivirus_segment 2	571bp
Af_PV_seg2-R	GCAACCAATTCGGGAGATGC		
Af_PV_seg3-F	ACCGTTATGGCAAGTACCCG	Detecting partitivirus_segment 3	542bp
Af_PV_seg3-R	AGGAACAGCATCTTTCGGCA		
Af_PV_seg4-F	GGAAGAAGCCCTGGACGAAA	Detecting partitivirus_segment 4	592bp
Af_PV_seg4-R	TGGGTGTTTGATCCTGCCAG		
Af_PV_seg5-F	ATGGTCAAGTCCCCTCGTCT	Detecting partitivirus_segment 5	542bp
Af_PV_seg5-R	GAAGGGTGGTGGTATGCGAT		
Af_PV_seg6-F	AACCGCCTACCAGATTTCGG	Detecting partitivirus_segment 6	509bp
Af_PV_seg6-R	GCTTCAACAACGGCAGACTG		
Af_PV_seg7-F	TTCGTCCGCAAGAACAAGGT	Detecting partitivirus_segment 7	526bp
Af_PV_seg7-R	GAGGCAACCAACGTAGACGA		
Af_PV_seg8-F	TGCTGTTTCCGTTGCAGGTA	Detecting partitivirus_segment 8	513bp
Af_PV_seg8-R	TTGTTGCCTCTATCGGGTGG		

Table S9 Genome sequencing data quality, related to "Whole genome sequencing" in STAR Method.

Sample	Raw reads	ount of data (	Effective(%)	Error(%)	Q20(%)	Q30(%)	GC(%)
IFM 65242	8311116	1.2	100	0.04	93.33	87.47	50.13
	29785026	4.5	100	0.03	97.46	93.61	48.69
IFM 65241	15465548	2.3	100	0.03	93.31	88.34	49.69
IFM 64473	17731626	2.7	100	0.03	94.44	89.03	49.7
IFM 63847	9402310	1.4	100	0.03	94.27	88.74	50.36
	26426836	4	100	0.03	96.99	92.7	48.68
IFM 63449g	15718694	2.4	100	0.03	95.62	90.79	48.53
IFM 63449w	8618454	1.3	100	0.03	93.76	88.65	48.5
	27538406	4.1	100	0.03	96.82	92.87	48.42
IFM 61879	6694780	1	100	0.03	95.23	90.27	47.73
	27053342	4.1	100	0.03	97.13	92.99	48.96
IFM 61226	10221012	1.5	100	0.03	95.45	90.39	47.45
	7467030	1.1	100	0.03	96.91	92.66	48.91
IFM 49866	14606140	2.2	100	0.03	95.75	90.75	48.31
NRRL3357	18369666	2.8	100	0.03	94.61	89.51	48.09

Table S10 RNA sequencing data quality, related to "RNA-sequencing analysis" in STAR Method.

Strain	Virus	Raw reads	Amount of data (Gb)	Effective(%)	Error(%)	Q20(%)	Q30(%)	GC(%)
IFM 65242	free	19229756	3.3	100	0.03	97.72	93.91	52.4
		3037422		100	0.03	97.73	93.73	52.38
		16483938	3.4	100	0.03	97.67	93.83	52.64
		6021860		100	0.03	97.71	93.68	52.64
		23968556	3.6	100	0.03	97.49	93.42	52.51
	P	22913634	3.4	100	0.02	98.09	94.59	52.42
IFM 65241	free	17734498	3.1	100	0.03	97.26	93.03	52.57
		2794068		100	0.03	97.37	92.98	52.6
	P	23493726	3.5	100	0.03	97.27	93.01	52.62
IFM 64473	free	30744022	4.6	100	0.03	97.51	93.38	52.61
	dF	16257998	2.4	100	0.03	97.46	93.2	52.39
IFM 63847	P/Pm	24199784	3.6	100	0.03	97.66	93.97	52
	Pm	19391324	2.9	100	0.03	97.86	94.33	52.19
	P	21112064	3.2	100	0.03	97.04	92.59	52.2
	free	17744916	2.7	100	0.03	97.13	92.75	52.03
IFM 63449g	free	17292230	2.6	100	0.03	97.07	92.62	52.15
	N	17715912	2.7	100	0.02	97.92	94.45	52.21
IFM 63449w	free	20982258	3.1	100	0.03	97.9	94.28	52.46
	N	16354188	2.5	100	0.03	97.85	94.17	52
IFM 61879	free	33563070	5	100	0.03	97.52	93.41	52.53
	V1	23257064	3.5	100	0.03	97.86	94.05	53.53
IFM 49866	free	29750968	4.5	100	0.03	97.58	93.46	52.56
	V2	31427170	4.7	100	0.03	97.53	93.36	52.23
Ha(IFM 65242)	free	22002764	3.3	100	0.03	97.69	93.9	52.64
	Va (IFM 65242)	34662920	5.2	99.9	0.03	96.59	91.56	52.55
		26261180	3.9	100	0.03	97.15	92.6	52.51
		24733724	3.7	99.91	0.03	96.84	91.95	52.49
	Vb (IFM 65241)	19249638	2.9	99.91	0.03	96.73	91.76	52.44
		26347464	4	100	0.03	96.5	91.31	52.47
		28880614	4.3	100	0.03	96.42	91.06	52.44
Hb (IFM 65241)	free	36378982	5.5	99.98	0.03	97.51	93.28	52.57
	Va (IFM 65242)	29664016	4.4	99.91	0.03	96.3	91.01	52.75
		32745874	4.9	99.9	0.03	96.65	91.67	52.91
		28637234	4.3	100	0.03	97.41	93.14	52.9
	Vb (IFM 65241)	29365686	4.4	100	0.03	96.97	92.39	52.96
		31038304	4.7	100	0.03	96.49	91.25	52.76
		36538214	5.5	99.98	0.03	97.77	93.84	52.47
